# Comparison of Random Forest Model and Frequency Ratio Model for Landslide Susceptibility Mapping (LSM) in Yunyang County (Chongqing, China)

**DOI:** 10.3390/ijerph17124206

**Published:** 2020-06-12

**Authors:** Yue Wang, Deliang Sun, Haijia Wen, Hong Zhang, Fengtai Zhang

**Affiliations:** 1 The Key Laboratory of GIS Application Research, Chongqing Normal University, Chongqing 401331, China; wwyue1998@aliyun.com (Y.W.); zh_angh@cqnu.edu.cn (H.Z.); 2 Key Laboratory of New Technology for Construction of Cities in Mountain Area, Ministry of Education, Chongqing 400045, China; 3 National Joint Engineering Research Center of Geohazards Prevention in the Reservoir Areas, Chongqing 400044, China; 4 School of Civil Engineering, Chongqing University, Chongqing 400045, China; 5 School of Management, Chongqing University of Technology, Chongqing 400054, China; zhfthero45@cqut.edu.cn

**Keywords:** Yunyang County, landslide susceptibility, random forest model, frequency ratio model

## Abstract

To compare the random forest (RF) model and the frequency ratio (FR) model for landslide susceptibility mapping (LSM), this research selected Yunyang Country as the study area for its frequent natural disasters; especially landslides. A landslide inventory was built by historical records; satellite images; and extensive field surveys. Subsequently; a geospatial database was established based on 987 historical landslides in the study area. Then; all the landslides were randomly divided into two datasets: 70% of them were used as the training dataset and 30% as the test dataset. Furthermore; under five primary conditioning factors (i.e., topography factors; geological factors; environmental factors; human engineering activities; and triggering factors), 22 secondary conditioning factors were selected to form an evaluation factor library for analyzing the landslide susceptibility. On this basis; the RF model training and the FR model mathematical analysis were performed; and the established models were used for the landslide susceptibility simulation in the entire area of Yunyang County. Next; based on the analysis results; the susceptibility maps were divided into five classes: very low; low; medium; high; and very high. In addition; the importance of conditioning factors was ranked and the influence of landslides was explored by using the RF model. The area under the curve (AUC) value of receiver operating characteristic (ROC) curve; precision; accuracy; and recall ratio were used to analyze the predictive ability of the above two LSM models. The results indicated a difference in the performances between the two models. The RF model (AUC = 0.988) performed better than the FR model (AUC = 0.716). Moreover; compared with the FR model; the RF model showed a higher coincidence degree between the areas in the high and the very low susceptibility classes; on the one hand; and the geographical spatial distribution of historical landslides; on the other hand. Therefore; it was concluded that the RF model was more suitable for landslide susceptibility evaluation in Yunyang County; because of its significant model performance; reliability; and stability. The outcome also provided a theoretical basis for application of machine learning techniques (e.g., RF) in landslide prevention; mitigation; and urban planning; so as to deliver an adequate response to the increasing demand for effective and low-cost tools in landslide susceptibility assessments.

## 1. Introduction

As frequently occurring geohazards in the world, landslides have features of slow movement but progressive deformation and destruction, often causing significantly severe damage in terms of losses both in human lives and properties. Landslides mainly develop in mountainous areas and cause serious threats to environments, settlements, and industrial facilities. In particular, after sliding into the river, landslides can block the river, form natural dams, and cause floods. In addition, they cause shipwrecks and casualties. On the other hand, landslides occurring in a reservoir can generate huge surges, turning over the dam and rushing downstream to destroy buildings, farmland, and roads. Sometimes, massive landslides can also trigger slight earthquakes. What is worse, it is not easy to implement monitoring and defense measures; thus, the losses tend to be extremely serious [1,2]. Seventy percent of China’s territory consists of mountainous areas, with extensive and highly frequent landslide disasters, and this situation becomes increasingly serious year by year. Among them, rainfall-induced landslides are the most widely distributed, with the highest occurrence frequency and the most serious damage. Over the past 60 years, the deaths caused by landslides in China have exceeded 25,000 persons, with an average of more than 400 deaths per year; economic losses are as high as US $50 million [3]. As the most typical mountain city in China, Chongqing is ranked first among the 70 cities with severe geological disasters such as collapses and landslides [4]. From 1950 to 2011 in Chongqing, there were a total of 16,554 recorded landslide disasters, with an average of 271 times per year [5]. The grim situation makes the measures to prevent and forecast landslide disasters extremely urgent. As one of such measures, landslide susceptibility mapping (LSM) usually serve as a foundation for landslide prevention and spatial planning because they depict the possibility of landslides in a region in the future based on the influence of terrain and environment, human activities, etc. [6]. It evaluates the geographical spatial distribution of potential landslide disasters by analyzing the internal and external factors affecting them, thus making potential disasters visible in space and providing a strong reference for relevant agencies to carry out preventive measures [7].

The effectiveness of LSM depends greatly on the modeling methodologies adopted. There are many methods for landslide susceptibility evaluation, such as qualitative, deterministic, statistical, machine learning, and other methods. In qualitative methods based on experience, engineering geologists and geomorphologists use expert experience and knowledge to directly or indirectly analyze and draw LSM on the existing topographic maps and engineering geological maps [8]. The disadvantage of such a method is its subjectivity and non-quantitative nature. The deterministic method is based on physical mechanics models for slope stability calculation by inputting the physical mechanic and hydrological parameters of soils, calculating the stability of areas in GIS (Geographic Information System) software, and finally outputting the LSM. Guimarūes et al. have researched LSM by using the deterministic method [9]. However, this method assumes that the parameters are in uniformity and the landslide surface consists of loose soil, leading to the fact that the calculated results are often quite different from the actual situation. On the other hand, various statistical analysis models had been widely used in the early stage, including frequency ratio (FR), weights-of-evidence, the analytical hierarchy process, evidential belief function, information model [10,11,12,13], etc. Generally, these methods become relatively mature in the field of landslide susceptibility. After comparing various statistical methods, it was found that the performance of FR model was generally better than others. For example, Wang et al. [10] compared the FR model and the index of entropy model, finding that, in terms of the success rate curve, the area under the curve (AUC) of FR and the index of entropy models were 0.8191 and 0.8109 for accuracy, respectively. Similarly, the prediction accuracy was 81.75% for the FR model and 81.44% for the index of entropy model. Bourenane et al. [14] compared five methods (FR, weighting factor, logistic regression, weights-of-evidence, and the analytical hierarchy process), concluding that the FR method can provide a more accurate prediction (86.59%), while the logistic regression model had the lowest accuracy (70.45%). Furthermore, as a bivariate statistical method, the FR model depends on the observed relationship between the distribution of landslides and each conditioning factor, and it is easy to implement and has accurate results. As a traditional method, the FR model may gradually fade with the rapid development of machine learning. However, it is widely used in landslide susceptibility evaluation [15,16] and has been proven effective. What is more, the input, output, and calculation process of the FR model are easy to understand, and even massive data can be processed quickly and easily in the GIS environment. 

With the development of geographic information systems and artificial intelligence, more and more studies begin to apply various machine learning methods, including logistic regression [17], decision tree [18], support vector machine [2], and so on. Although these methods have been widely used in the field of landslides, they have certain deficiencies, such as complex modeling processes, unstable model performance, and weak interpretation [19]. To avoid such problems, the random forest (RF) model is proposed in combination with multiple decision trees to improve prediction accuracy, and the model’s output is determined by the modes of various trees. This model can handle high-dimensional and large data sets, with strong generalization ability, thus superior to traditional methods such as logistic regression [20]. Compared with its application in other fields [21,22,23,24,25,26], the RF model has just begun to be used in landslides over recent years [27,28]. Li et al. [29] applied the RF model in landslide disaster susceptibility mapping and factor evaluation. Yu et al. [30] applied this model in an empirical analysis of the relationship between landslide occurrence and landslide factors in Fujian Province of China and explored its adaptability in spatial prediction of landslides in southern China. They have achieved good landslide susceptibility model performance. 

In fact, due to that the occurrence of landslides is closely related to geological features, crustal movement and human activities, etc., landslides are affected by multiple factors at varying degrees. Therefore, they have the properties of imbalance, nonlinearity, multi-scale, randomness, etc., which have not been systematically resolved in regional landslide susceptibility evaluation researches [31]. Each method has its advantages and disadvantages, and in general, their performance depends on the differences of the research areas and the factors selected. Hence, it is meaningful to use different methods to compare the landslide susceptibility in researches. Although many studies have compared various methods, few have compared machine learning and statistical analysis methods. 

This study analyzed landslide susceptibility by using the RF model, which performs better as compared with machine learning methods, and the FR model, which also has had good results as compared with other statistical methods. Although the two methods are different in their algorithm, they each have their own advantages and can achieve good results. Therefore, this study compares the differences between data mining techniques and bivariate statistical analysis methods based on LSM acquired from the RF model and the FR model, and the study results can represent a theoretical and practical guidance to method selection. The evaluation of landslide susceptibility depends on the regional differences, the amount of data, and the accuracy of conditions. Selecting appropriate models and conditioning factors can facilitate satisfactory results. Previous studies proposed many factors for evaluating the susceptibility of landslides, but less consideration was given to human activities and soil erosion factors. 

As a typical karst area in the Three Gorges Reservoir area, Yunyang County faces serious soil erosion, while human construction activities are also important factors inducing landslides [32,33]. Therefore, based on the existing literature, 22 conditioning factors in five types (namely, topography factors, geological factors, environmental factors, human engineering activities, and triggering factors) were selected to evaluate the LSM. Specifically, this study added the POI (point of interest) kernel density, sediment transport index (STI) [34], stream power index (SPI) [35], and terrain roughness index (TRI) [36] as factors affecting landslide susceptibility, and then established a landslide susceptibility spatial database. POI kernel density has not been used in previous studies. As one of the important factors in human engineering activities, it affects the occurrence of landslides. The advantages and disadvantages of the two models were comprehensively explored, and their results were validated and compared by using receiver operating characteristic (ROC), the area under the curve (AUC) value of ROC curve, precision, accuracy, and recall ratio. Finally, the distribution characteristics of new landslide events and the importance of landslide-influencing factors were analyzed, aiming to provide a reference for landslide disaster prevention and urban construction planning. 

## 2. Materials and Methods 

### 2.1. The Study Area

**Figure 1:**
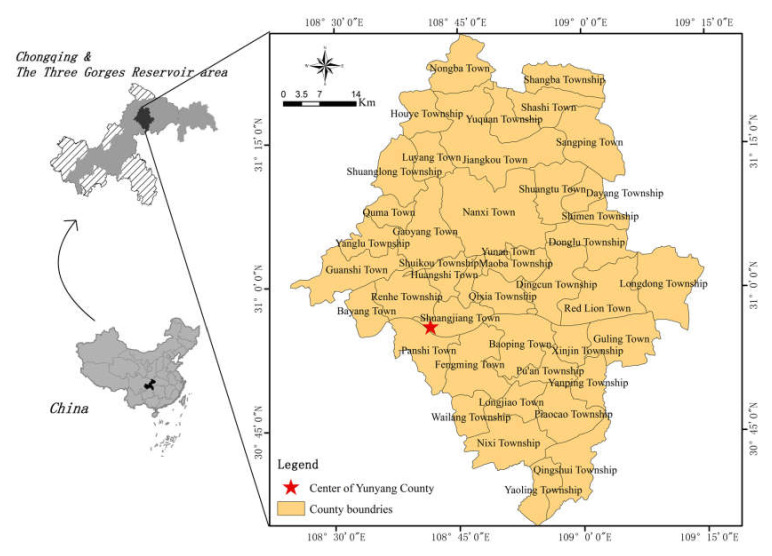
Location of the study area.

Yunyang County, the study area, is located in the northeast of Chongqing, the center of the Three Gorges Reservoir area, with an area of 3649 km^2^ (Figure 1). Also located in the eastern edge of Sichuan Province, the study area is controlled by Huaying Mountain-Fangdou Mountain and Daba Mountain curved fold systems, featuring a folded geological structure. There are no large-scale fault structures in the study area, generally with only small-scale faults on rock surfaces [37]. As a transition zone from hills to mountains, the study area has high elevations in its southern and northern parts and low elevations in its middle part, with large topographic undulation. 

**Figure 2:**
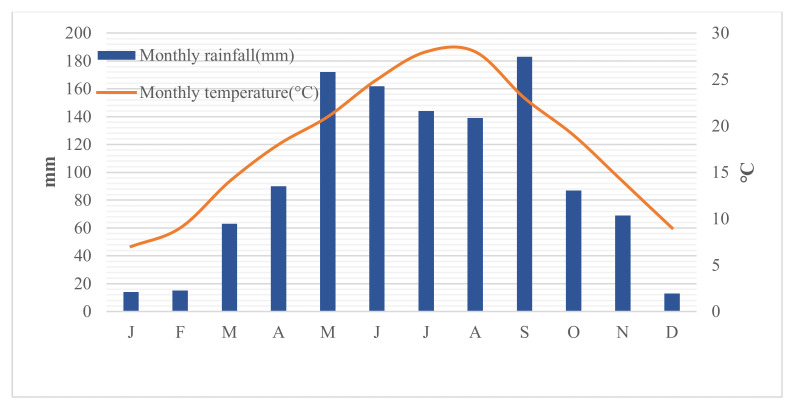
Rainfall and temperature distribution in Yunyang County (2009–2018).

Its climate is the subtropical monsoon, with hot and rainy summers and relatively warm and rainless winter, showing four distinct seasons. The average monthly temperature is 7 °C (January) to 28 °C (August and September). The highest temperatures are characterized by simultaneously extremely rainy and hot days. On the other hand, the study area is abundant with rainfall, with an annual average rainfall of 1151 mm. Its precipitation mainly occurs from May to September, with 73.1% of the annual rainfall mostly related to thunderstorms (Figure 2). The surface water system in the area is well developed, as the Yangtze River, dozens of its tributaries and many small creeks constitute a complex surface runoff system. 

Yunyang County is in a karst landform. Its strata include Permian, Triassic, Jurassic, and Quaternary formations. Limestone was widely formed in the Permian and Triassic periods, followed by sandstone and mudstone in the Jurassic. In addition to dissolution, such mechanical erosions as water erosion, rainfall erosion, and wind erosion aggravate the soil and water loss in the study area, providing developmental conditions for landslides. Human engineering activities, such as town construction, resettlement, water storage, and power generation in the reservoir areas, as well as continuous precipitations, lead to frequent landslide disasters; moreover, the large scale, variety, and wide range of landslides cause huge loss of life and property [38]. After investigation, it was found that there are geological disasters such as landslides, mudflows, and collapses, and especially 987 landslides, accounting for 97.3% of all geological disasters in Yunyang County. Although relevant agencies investigate, monitor, and issue early warnings for very large landslides, new landslide disasters occur every year in Yunyang County under the influence of heavy rains in the flood season and the changed water levels due to the Three Gorges Reservoir. Because these disasters are not monitored by the National Land Ministry, how to avoid and prevent geological disasters has become a key issue for local governments. 

### 2.2. Datasets

#### 2.2.1. Landslide Inventory

**Figure 3:**
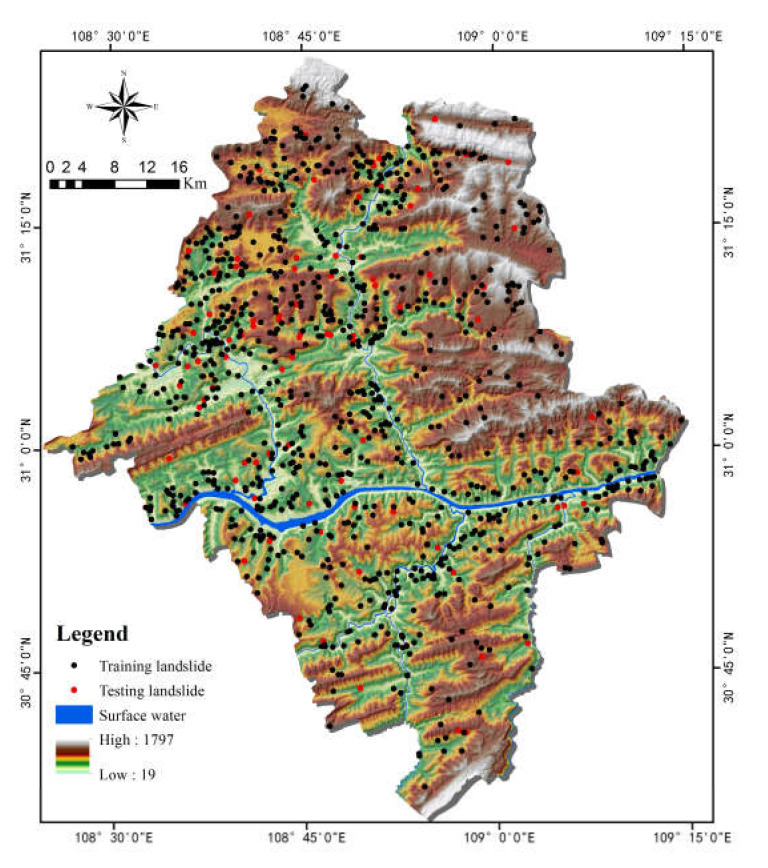
Landslide Inventory Map of the study area.

**Figure 4:**
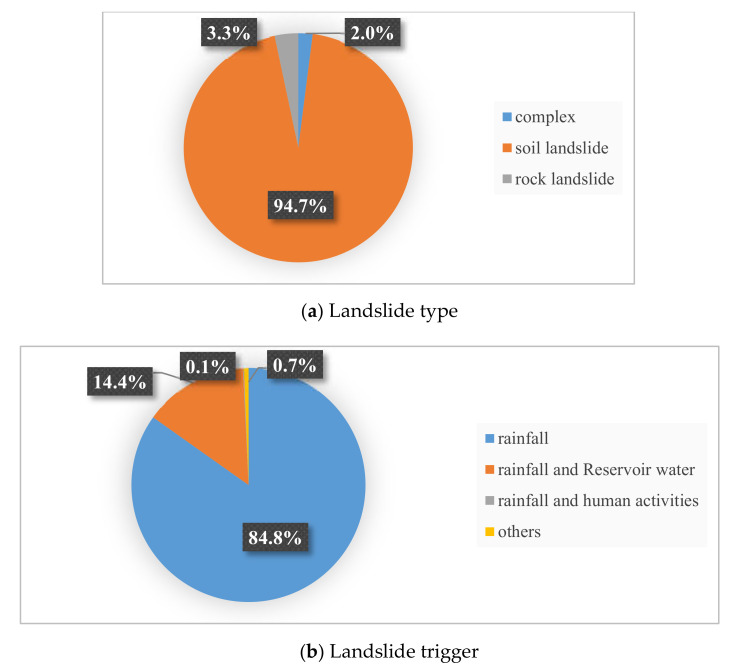
The scale of landslide type and trigger.

A landslide inventory map records the location, where known, the date of occurrence, and other information about landslides in an area [39]. Preparation of landslide inventory constitutes an essential basis for assessing the landslide susceptibility in this study [40,41], which identified and mapped a total of 987 landslides locations (2001–2016) (Figure 3) based on historical records, satellite images and extensive field surveys, thus building up the landslide inventory. In Figure 3, landslides for training and testing are marked in different colors by GIS software. The smallest landslide area is 100 m^2^, while the largest is 3,200,000 m^2^, averaged at 95,127 m^2^. Hungr et al. [42] increased the 29 landslide types of Cruden et al. [43] to 32. Although a clear-cut boundary for landslide classification is always controversial, it still has important guiding significance for landslide research. Given the actual situations of the landslides in the study area, they were sorted out on two bases, i.e., type and trigger, according to their material composition and inducing factors (Figure 4). In terms of type, it can be found that most of the landslides in the study area were soil ones (94.7%), while rock landslides and complex landslides (soil and rock landslides) only accounted for 3.3% and 2.0%, respectively. Although complex landslides were not included in the classification made by the above researchers, they can be described by using a combination of two or more types of landslides. On the other hand, many scholars suggested that different types of landslides should be treated separately; however, soil landslides were most frequently seen in the study area, and different types of landslides have been proven to be effective in the same method [44,45]. The landslide area is mainly distributed with residual, slope soil and mudstone, shale or siltstone, etc., without active faults. There are mostly small and medium overburden soil landslides, most of which are developed in low mountain areas and hilly landform with a slope of 20–40 degree, and the stability is poor. The bedrock of the overburden landslides is mainly J2s or J3s strata. The materials of the landslides are primarily composed of purple or brown silty clay and gravel. The above materials contribute to the penetration of surface water, and are easily softened in water, which is an important internal cause of landslide deformation. In terms of trigger, most of the landslides in the study area were caused by rainfall (84.8%), rainfall and Reservoir water (14.4%), coupling (0.7%), and human engineering activities (0.1%, only one). Therefore, rainfall is the main trigger of landslides in Yunyang County. 

**Figure 5:**
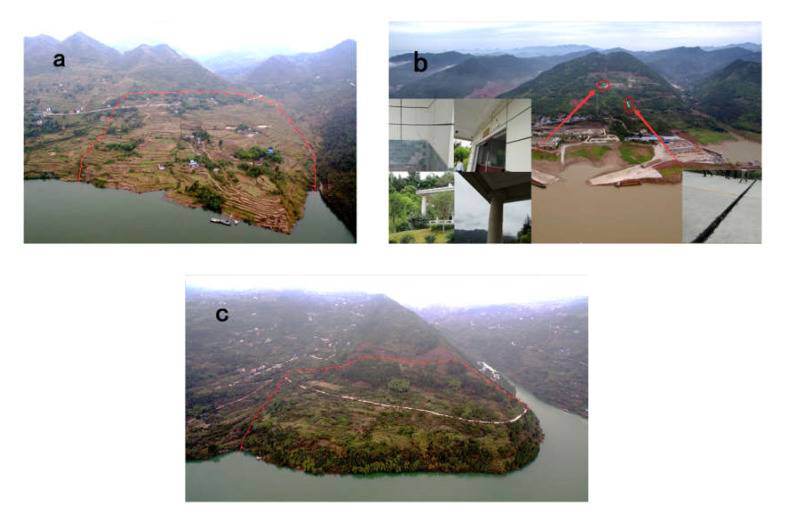
Typical landslides: (**a**) Dashiban Landslide (1 April 2014); (**b**) Jiuxingping Landslide (15 June 2012); and (**c**) Liekou Mountain Landslide (1 April 2014).

Typical landslides in Yunyang County, such as the Dashiba Landslide, Jiuxianping Landslide, and Liekou Mountain Landslide (Figure 5a–c), are located around reservoirs. The Dashiban Landslide and Liekou Mountain Landslide occurred on 1 April 2014 (in spring), and the Jiuxingping Landslide occurred on 15 June 2012 (in summer). Due to the rainy spring and summer in the study area, numerous fissures favored by the strong permeability of limestone and clays enable rainfall infiltration, river erosion, and rock spreading [46]. The Dashiba Landslide is a soil landslide with a volume of 1.365 × 10^8^ km^3^, resulting in 432 people injured and a direct economic loss of up to 20.4 million CNY. The Jiuxianping Landslide is a rock landslide, still in poor stability currently, causing a new highway to have obvious and widening cracks. In addition, a crematorium and a cemetery were evidently deformed (Figure 5b). The volume of the landslide is 2.7 × 10^6^ km^3^, causing property damage of up to 16 million CNY. The Liekoushan Landslide is a mixed one, combining the features of bedrock and accumulated-layer landslides, with a volume of 1.342 × 10^6^ km^3^, causing property damage of 450,000 CNY. The analysis of typical landslides is helpful to understand the specific situation of the landslide in the study area. Subsequently, the new landslides in 2017 will also be considered in Section 4.2 for accuracy evaluation of the RF and FR model.

#### 2.2.2. Conditioning Factors of Landslides

The development of landslide disasters is not only controlled by the geological conditions of the slopes, but also by external factors such as hydrological conditions, climatic conditions, and human engineering activities [47]. Reichenbach et al. [48] identified 596 factors for landslide susceptibility and classified the factors into five types: geology, hydrology, land cover, landforms, and others. The selection of conditioning factors as input variables in models is a crucial step in LSM. Based on the existing research results and literature [49,50] as well as the overall characteristics of landslide development in Yunyang County, this study selected 22 secondary conditioning factors under five primary conditioning factors to construct a basic evaluation system for landslide susceptibility. The acquisition path and classification of conditioning factor is directly related to their different natures [51], as described on Table 1.

##### Topographic Factors

**Figure 6:**
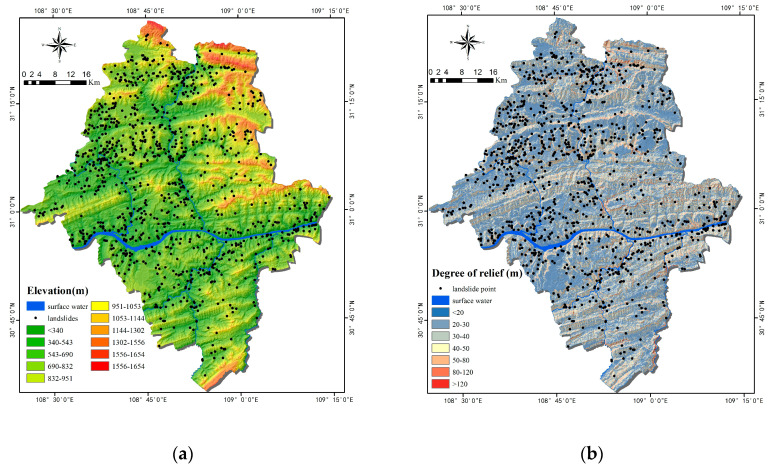

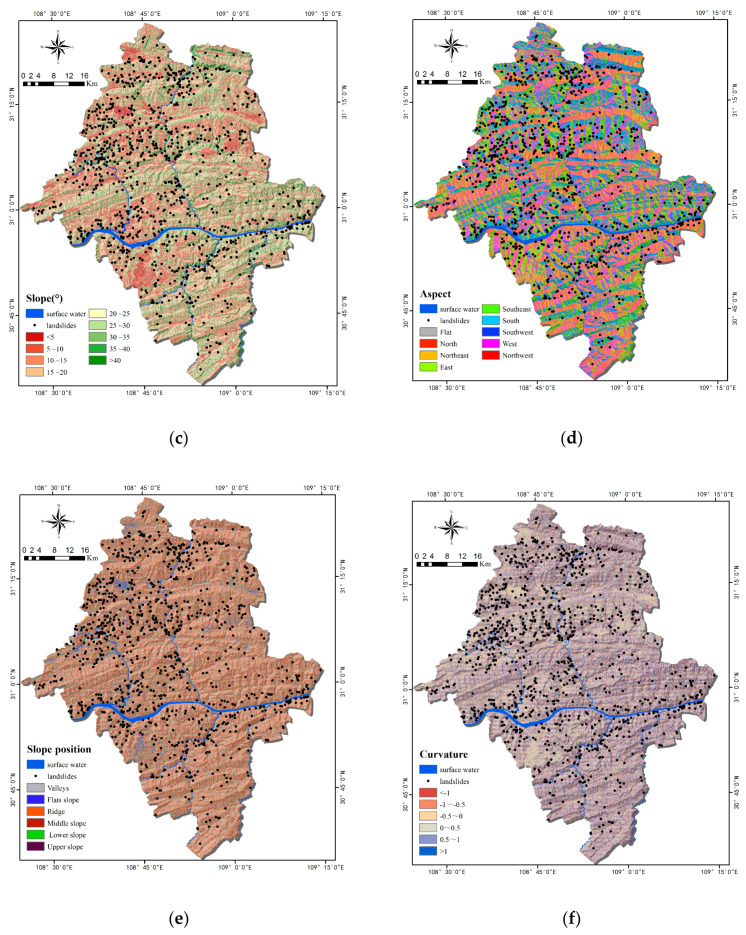

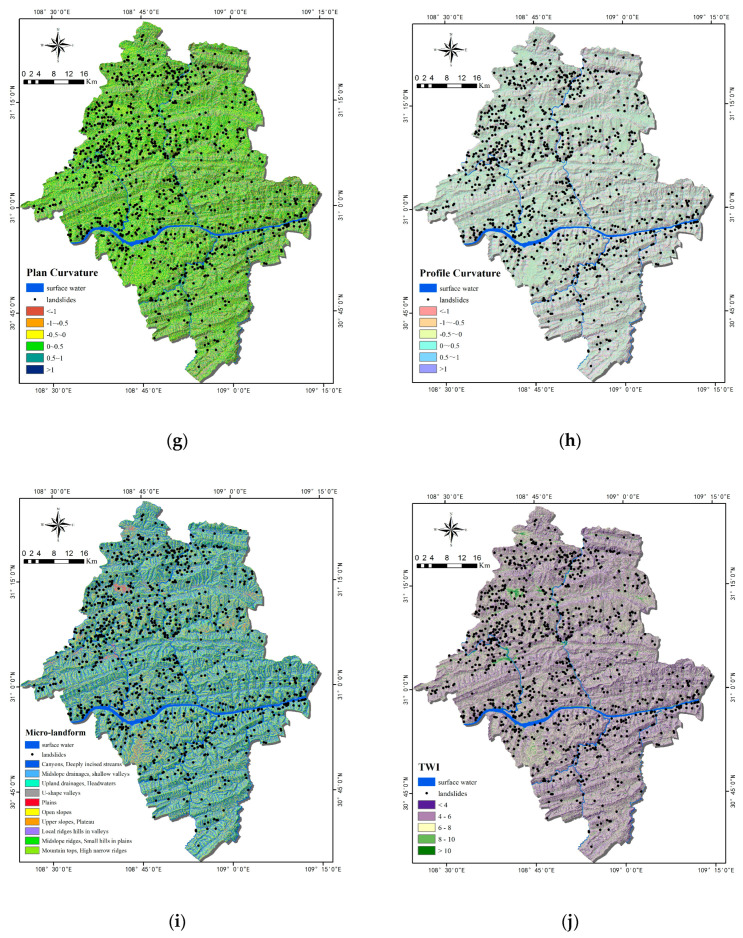

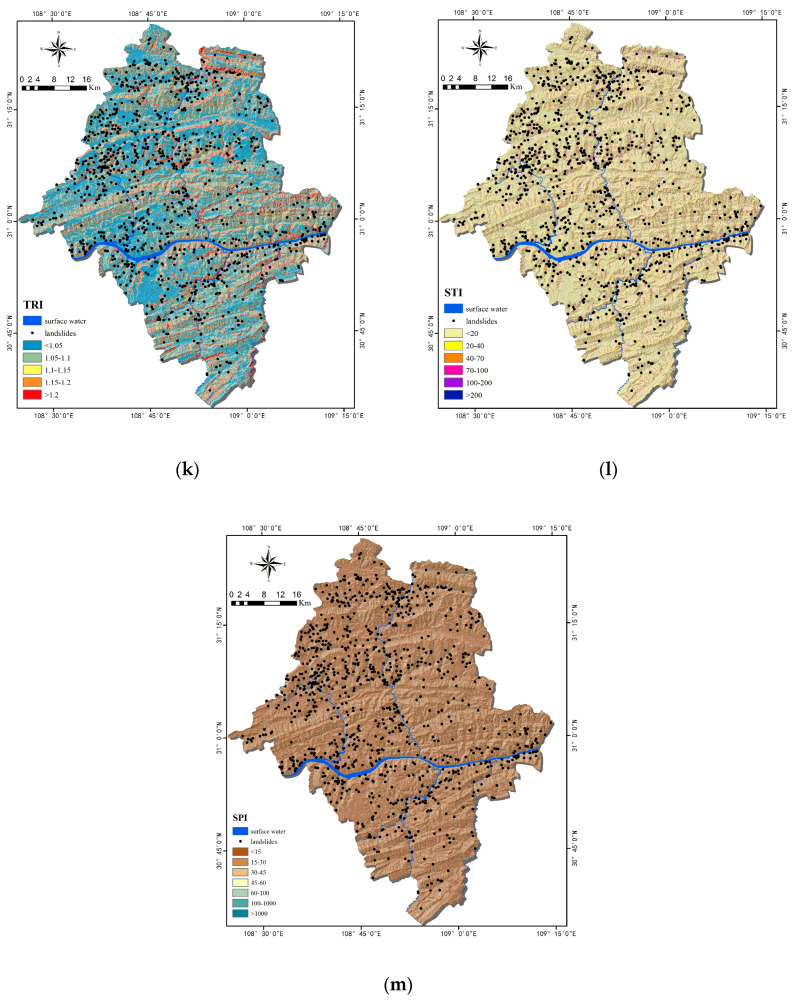
Thematic maps of topographic factors: (**a**) Elevation; (**b**) relief degree of land surface (RDLS); (**c**) Slope; (**d**) Aspect; (**e**) Slope position; (**f**) Curvature; (**g**) Plan Curvature; (**h**) Profile Curvature; (**i**) Micro-landform; (**j**) topographic wetness index (TWI); (**k**) terrain roughness index (TRI); (**l**) sediment transport index (STI); (**m**) stream power index (SPI).

Topographic factors include elevation, relief degree of land surface (RDLS), slope, aspect, slope position, curvature, plan curvature, profile curvature, micro-landform, topographic wetness index (TWI) terrain roughness index (TRI) sediment transport index (STI) and stream power index (SPI). These factors were all calculated with a digital elevation model (DEM). Aspect, slope position, and micro-landform are categorical factors, and thus, should be separated first (Table 1). Others are continuous factors and should be discretized. The conventional factors include: elevation, RDLS, slope, aspect, slope position, curvature, plane curvature, and profile curvature (Figure 6a–h). Micro-landform is a relatively small geomorphology unit, and there exist 10 types, including canyons, deeply incised streams, mid-slope drainages, etc. (Figure 6i). TWI, TRI, STI and SPI were extracted with ArcGIS 10.4 software. TWI defines the amount of water flow accumulated at any site in a catchment and the ability of the water to flow downward under gravity (Figure 6j). TRI map and STI map (Figure 6k,l) were prepared and divided into five subclasses and six subclasses, respectively. SPI is the power of water flows in cases of erosion (Figure 6m). TWI, TRI, SPI and STI are defined as:

(1) $$TWI=\log_{e}(\frac{A}{\tan\beta })$$

(2) TRI=1/cos(tanβ⋅π/180)

(3) SPI=A⋅tanβ

(4) $$STI={\left(\frac{A}{22.13}\right)}^{0.6}\times {\left(\frac{\sin\beta }{0.0896}\right)}^{1.3}$$

where *A* is the flow accumulation in square meters (m^2^/m) and *β* is the slope (in degrees).

##### Geological Factors

**Figure 7:**
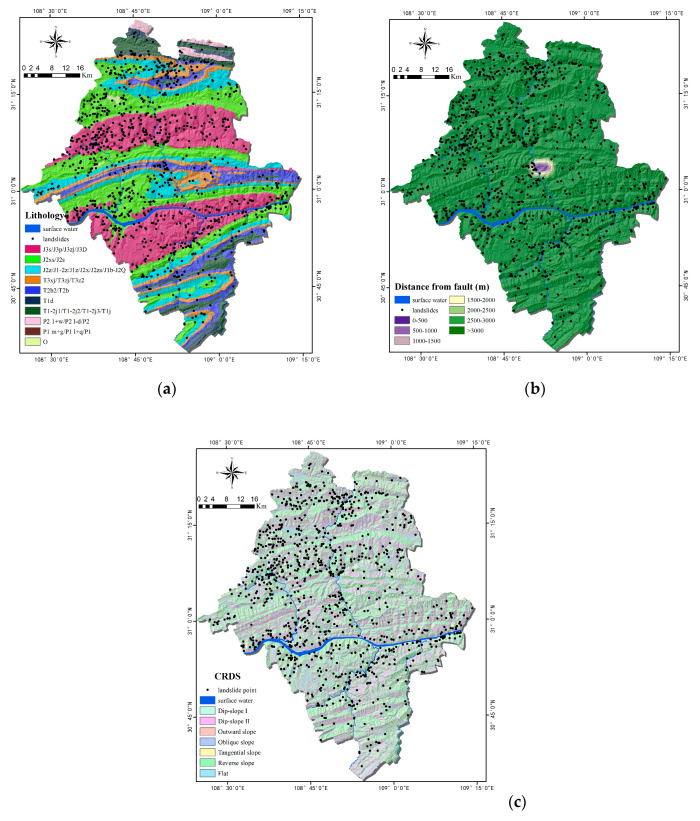
Thematic maps of geological factors: (**a**) Lithology; (**b**) Distance from fault; (**c**) The combination reclassification of stratum dip direction and slope aspect (CRDS).

Three factors were used as geological factors: lithology, distance from fault, and combination reclassification of stratum dip direction and slope aspect (CRDS). As an important internal cause of landslides, different lithology features have large differences in physical and mechanical parameters and directly affect the slope stability. The lithology of Yunyang County (Figure 7a) mainly includes J2 strata (J2xs, J2s, j2z, J1-2z, J1z, J2x, and J2zs) and J3 (J3p, J3s, J3zj, and J3D). The study area’s fault scales are small, with no large fault zones (Figure 7b). CRDS (Figure 7c) is a preliminary evaluation method of slope stability based on topographic maps and geological maps, and it was classified into seven types [52]. 

##### Environmental Factors

**Figure 8:**
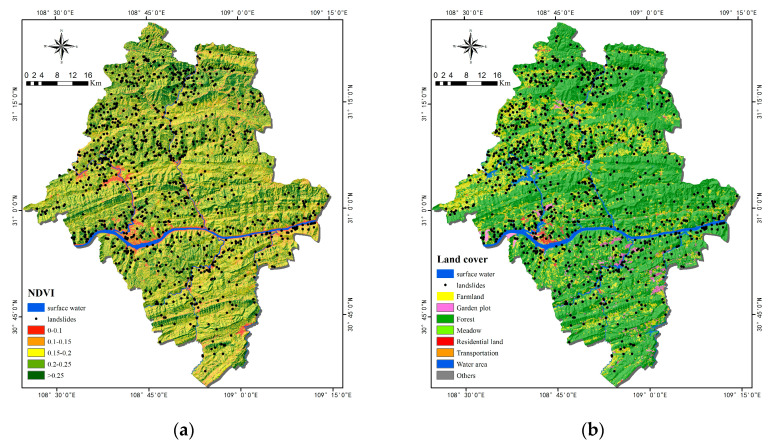
Thematic maps of environmental factors: (**a**) normalized vegetation index (NDVI); (**b**) Land cover.

As external factors affecting landslides, the normalized vegetation index (NDVI) and land cover are environmental factors. A positive NDVI value refers coverage of active forests or other vegetation biomasses. According to the NDVI values of the study area, its landslides generally occur in bare soils and grasslands. NDVI was classified into five subclasses (Figure 8a). Land cover is widely considered as an important factor in small and medium landslides. In fact, the roots of vegetation can reinforce the soil and increase soil shear strength. There are many types of land cover in the study area, and they were used as factors for landslides (Figure 8b). 

##### Triggering Factors

**Figure 9:**
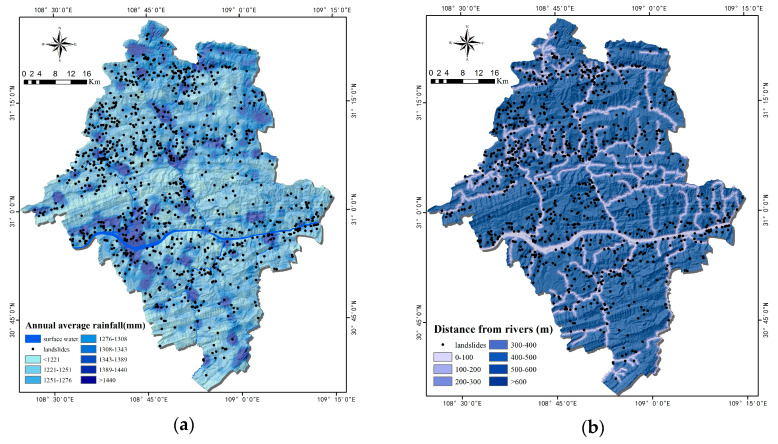
Thematic maps of triggering factors: (**a**) Annual average rainfall; (**b**) Distance from rivers.

Rainfalls and distances from rivers are the main trigger factors in the area. In total, 84.8% of its landslides are caused by rainfall. There exists uneven distribution of rainfall in Yunyang County (Figure 9a). The surface water formed by rainfall would not only wash the slope surfaces, but also infiltrate and soften the rocks and soils. In consequence, the slippage-resisting ability of slopes is reduced. Riverbank erosion is another essential cause of landslides [53]. Owing to bank cutting and toe erosion, slope bodies near rivers are prone to landslides. Chen et al. [54] applied an equal interval method to rivers, and therefore, it is categorized to equally partitioned segments. Hong et al. [34] performed the similar work that they use 200 m interval to produce the river buffer zones. The result shows that the higher correlation between landslides and the rivers within 200 m. Figure 9b shows most of the historical landslides are distributed along the rivers. The distance from the rivers was divided into seven classes. The density of the landslide is the highest within 200 m from the rivers, which is the main range of reservoir water to impact on landslides. Because the study area is in the center of the Three Gorges Reservoir, and the periodic rise and fall of the water level is one of the main causes of landslides. The water level in the reservoir fluctuate repeatedly from 145 to 175 m, forming a riparian zone with a height difference of 30 m. Chongqing’s riparian zone has an area of 306.3 km^2^ and the coastline is 4881.4 km. Meanwhile, Yunyang County is one of the four counties with the largest riparian zone. Long-term and periodic ups and downs of the reservoir water level caused the water flow to wash away a lot of soil, the river bank to become steeper, the gravity of the front edge of the landslide to decrease, and the supporting force of the front edge of the landslide to decrease, resulting in the decrease in the stability of the landslide. In particular, the slope bank was soaked for a long time by the reservoir water, which caused the soil to become soft. Additionally, during the fall of the reservoir water level (January to May), groundwater level drops slower than reservoir level, which increased sliding force of landslides significantly, after which the landslide occurred.

##### Factors of Human Engineering Activities 

**Figure 10:**
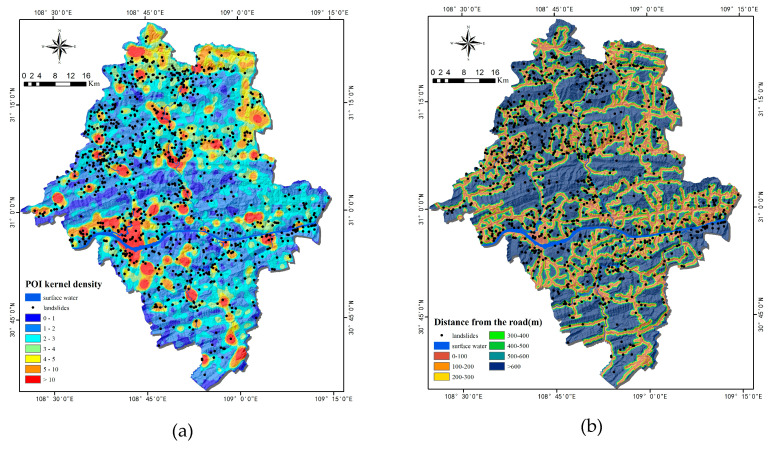
Thematic maps of human engineering activities: (**a**) Point of interest (POI) kernel density; (**b**) Distance from the road.

Factors of POI kernel densities and distance from roads are related to human engineering activities. POI is based on location services and usually contains the name, address, longitude, latitude, category, etc. If each POI site is regarded as a functional unit, then the higher the POI density, the more concentrated the urban functions in an area. POI kernel density analysis was made with ArcGIS software, which is often used to identify urban centers, economic vitalities, and intensities of human activities [55,56,57]. However, in the field of LSM, no research has been found when using POI as an influencing factor in human engineering activities thus far. The construction of massive roads is a process where human transform the natural environment, which includes transportation, erosion, and accumulation of surface soil. Excessive digging, application of external loads, and vegetation destruction lead to steep slopes and loose soil. Finally, precipitation and earthquakes can trigger landslides. Wen et al. [58] used 100 m as the road buffer interval in the region along the highway of Mao County. Bourenane et al. [59] made a bivariate statistical and expert approaches of the landslides in the city of Constantine, Algeria. They indicated that the main range to affect landslides was within less than 200 m from the roads. Therefore, we categorized the road at equal intervals (Figure 10b). According to the statistics, the landslide density is the largest within 200 m from the roads in the study area. As the buffer zone increases, the landslide density shows a decreasing trend, which shows that the main range of landslide occurrence is within 200 m of the roads in Yunyang County. 

Table 2 shows the data and their sources, types, and accuracy for the above 22 secondary conditioning factors. Historical landslide data and related geography, topographical geology, POI, and other data come from the years between 2001 and 2016, and they have temporal and spatial consistency with historical landslides. 

In summary, based on the investigation, extensive literature review and manual classification (expert experience), factors were categorized. All conditioning factors were converted into 30 m × 30 m grid units to establish a geospatial database of landslide conditioning factors. The common unit types used in LSM include grid units, drainage basin units, slope units, etc. Except for the first type, which is a regular unit, the others are irregular units. As the length and width of the landslides are relatively small, the grid units are the most prevalent method to represent the datasets of landslides [60]. On the contrary, it is better to use drainage basin units or slope units to evaluate mudflows, as they are mostly narrow and long.

In order to reduce the data dispersion, all the 22 factors after reclassification should be normalized. Among them, qualitative data such as lithology, land cover, slope position, micro-landform, aspect, and CRDS are divided into different classes before normalization. Then, the factors were transformed linearly after assigning an integer value (Starting from 1) to each class, so that their values were reduced to the [0, 1] interval. The normalization formula is:

(5) $$X^{*}=\frac{X-X_{min}}{X_{max}-X_{min}}$$

where X* is the normalized data; *X* is the original data; $X_{min}$ is the minimum value after each factor is assigned; and $X_{max}$ is the maximum value after each factor is assigned. 

### 2.3. Methodology

**Figure 11:**
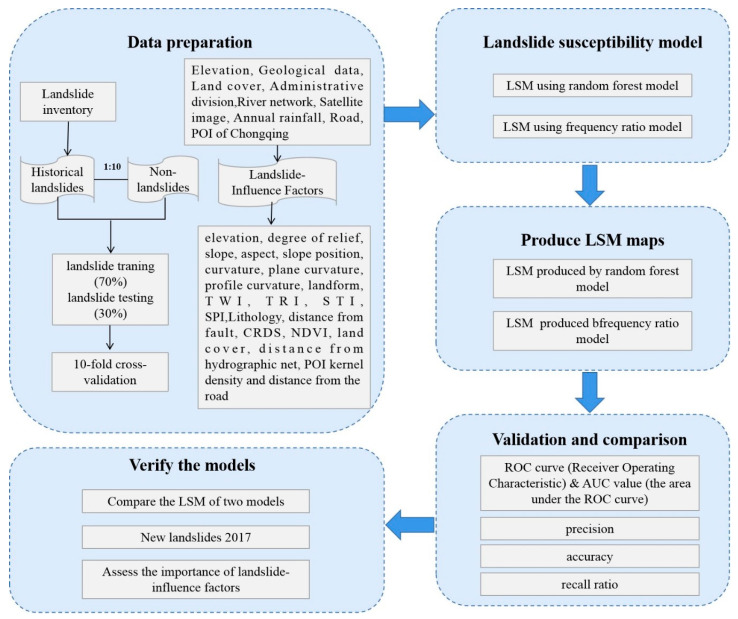
The methodological framework of the study.

This study is purposed to compare the machine learning method and statistical analysis method for LSM. The methodological framework of this study mainly includes five parts, as shown in Figure 11: (1) data preparation, including: collection of information on historical landslides and non-landslides, preparation of training and testing datasets before 10-fold-cross validation, and selection the landslide-conditioning factors; (2) landslide susceptibility modeling by using the RF model and the FR model; (3) drawing LSM maps by using the two models; (4) validation and comparison, covering: the ROC curve, AUC value (the area under the ROC curve), precision, accuracy, and recall ratio; and (5) verification and comparison of the two models, the new landslides in 2017, and the importance of the conditioning factors.

#### 2.3.1. Preparation of the Training and Testing Datasets 

LSM with the RF model (Machine learning) can be considered a binary classification. First and foremost, an adequate number of valid samples (landslide and non-landslide data) are extremely important. Especially, in machine learning methods, adequate data are needed to ensure a high learning performance. Because the information on historical landslides is limited in the study area, selecting more non-landslides could expend the quantity of sample data. On the other hand, based on previous researches of other scholars [61], when the ratio of positive samples to negative samples is 1:10, the model would deliver a better prediction performance. Hence, 987 positive samples and 9870 negative samples were collected into a dataset in this study. Moreover, in order to select the “non-landslide area” as widely as possible, this study considered the area excluding the 500 m buffer zone of all landslides and excluding the part where the rivers in the study area are located is taken as the non-landslide area.

Overfitting is a problem that cannot be ignored in RF model, although the model has good performance when dealing with big data. A very useful technique for testing and avoiding overfitting is cross-validation (Rotation Estimation). The data set was randomly divided into two subsets: 70% of the samples were used as training and 30% for testing. In order to get a reliable and stable model, the datasets were divided into ten independent subsets that all included 70% training set and 30% testing set (so-called 10-fold cross-validation). The random forest function in R studio software was used to develop the RF model with the training dataset.

#### 2.3.2. Random Forest (RF)

By building multiple decision trees from different subsets of data, RF is an integrated method that combines the ideas proposed by Breiman [62] and the methods described by Ho [63]. Compared with the traditional landslide division methods, the RF method introduces two random samplings (samples and features). The decision trees improve the accuracy and stability of the model better than a single decision tree by using a randomly generated method to select samples and features. Then, the judgment results of multiple decision trees are voted to get the final output. Many studies have shown that the RF has high tolerance in terms of algorithms, outliers, and noises [64] and can process multi-dimensional data without feature selection, with an easy implementation process in parallel. In this study, the RF consists of two trees (landslide and non-landslide), and each is constructed by using 22 random features.

The key point of RF is to combine n independent decisions $\left[y\left(X,\theta_{k};k=1,2,\ldots n\right)\right]$ to build a model. Each decision tree in the model judges or predicts the samples. Different classification models $y_{1}\left(X\right),\;y_{2}\left(X\right),\ldots ,y_{k}\left(X\right)$ are obtained after samples training. Then, these classification models can be used to build RF models:

(6) $$Y\left(X\right)=arg_{Z}^{max}\overset{k}{\underset{i=1}{\sum }}I\left(y_{i}\left(X\right)=Z\right)$$

where $Y\left(X\right)$ represents an RF model, $y_{i}\left(X\right)$ denotes a single decision tree model, Z means output variable, and $I\left(.\right)$ is an explicit function. 

**Figure 12:**
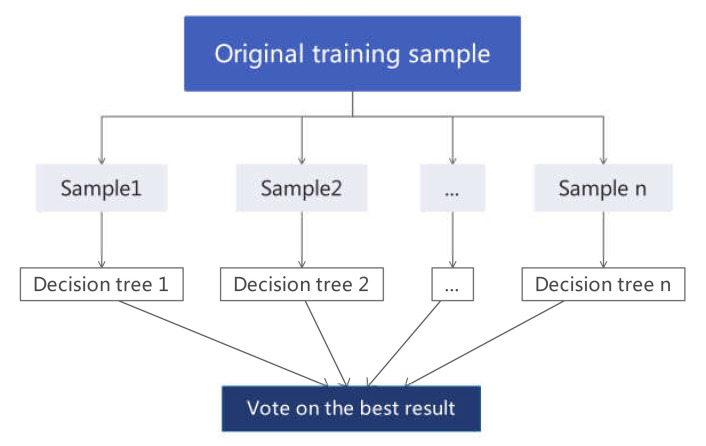
The schematic diagram of the RF algorithm.

Figure 12 shows the steps of the RF algorithm.

**Figure 13:**
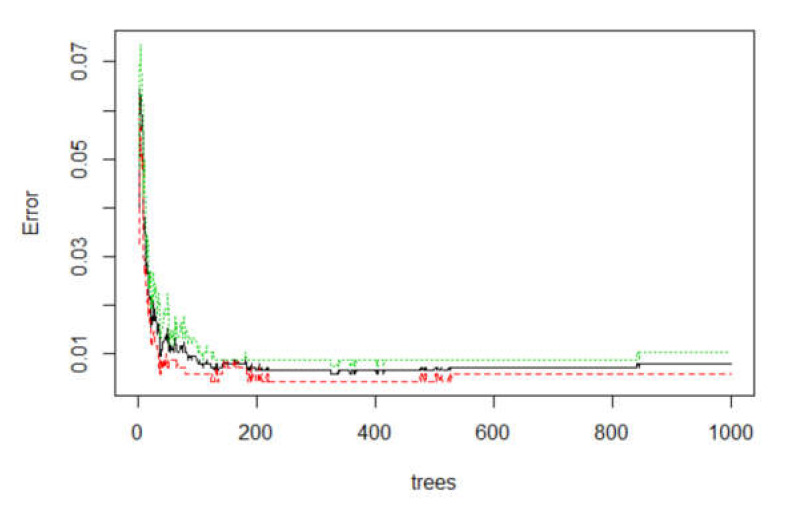
The error rate of the overall RF model (black line: OOB (out of the bag); red line: without landslide; green line: with landslide).

The procedure of RF is summarized as follows: (a) Determine the value of mtry i.e., to generate mtry variables for the binary tree on the nodes randomly. The choice of binary-tree variables needs to meet the principle of the minimum impurity. (b) On the one hand, the model uses the bootstrap method to randomly select ntree sample sets in the original data set to form ntree decision trees. On the other hand, unsampled samples are used for the prediction of a single decision tree. (c) ntree decision trees constitute a RF model, and then, the samples are predicted or classified based on the generated RF. The principle of classification is voting and the principle of prediction is a simple average.

mtry and ntree are two main parameters in RF model. The mtry parameter refers to the number of variables used in each decision tree, while ntree refers to the number of trees that the random forest contains [65]. Generally, mtry has a default of 2. On the other hand, it is also equal to the square root (classification model) or one-third (regression model) of the number of variables. Therefore, this study set mtry to 7. After solving the mtry’s value, it is brought into the RF model for training. While the out-of-bag (OOB) error is stable, the minimum value of the abscissa is ntree. In Figure 13, the proportion of misclassifications over all out-of-bag elements is the out-of-bag (OOB) error, which is an unbiased estimate of the generalization error. As the number of trees increases, the generalization error is always becoming steady. Hence, when trees are close to 860, the OOB error of the model tends to be stable. This study set ntree to 860.

In the process of building decision trees, this study uses the Classification and Regression Tree (CART) algorithm to split the nodes. CART follows the minimum principle of Gini. At node t, CART randomly extracts object is assigned to class i according to probability p(j|t). The estimated probability that the object belongs to class j is p(j|t). Under this rule, the estimated probability of misclassification is:

(7) $$Gini=\sum_{i\neq j}^{J}(p(i|t)p(j|t)$$

#### 2.3.3. Frequency Ratio (FR)

The FR model is based on the classification of certain conditioning factor states and calculates the degree of influence of each level state on landslides [15], which is a statistical analysis method based on susceptibility evaluation. The FR is defined as the ratio of the probability of the occurrence of landslides to the probability of non-landslide in given area. The model deduces the spatial relationship between the landslide occurrence locations and various factors affecting the landslide occurrence, improves the accuracy of state classification, and reveals the correlation between the landslide locations and various factors in the study area [66]. The frequency ratio $\left(Fr_{i}\right)$ assesses the relative importance of each class with respect to landslides. In order to implement the FR method, this study converted each factor to different classes (Table 6), and the Arc GIS software was used to produce the number of cells (for landslides and non-landslides) and the value of FR, which is defined as:

(8) $$Fr_{i}=\frac{b}{a}=\frac{\frac{landslide\_cell_{i}}{landslide\_cell_{tot}}}{\frac{no\_lanslide\_cell_{i}}{no\_lanslide\_cell_{tot}}}$$

The $Fr_{i}$ index indicates the importance of the states of the conditioning factors for the occurrence of landslides: FR>1 indicates that the state has a high correlation with landslide occurrence, and FR<1 indicates a low correlation.

The summation of each factor’s ratio was used to calculate the landslide susceptibility index (LSI)

(9) $$LSI=\sum Fr_{i}$$

where $Fr_{i}$ is the FR of each factor’s class (i=1,2,3…) and LSI is an index of the entire study area’s landslide.

#### 2.3.4. Evaluation of LSM Models

Model evaluation is the key to reflect model performance, and different aspects can be assessed for a model. The accuracy, precision (positive predictive value), sensitivity (true positive rate), and specificity are usually considered effective indicators of the fitting and predictive accuracies. Therefore, this paper applied these indicators to evaluate and compare the performances of the two models in the present research (Table 3).

Moreover, the ROC curve is also a method to measure the effectiveness of a model. The AUC value is used as the basis for determination [67]. This value ranges from 0.5 (very poor performance) to 1.0 (perfect performance). When the AUC value is greater than 0.7, the closer it is to 1, the more accurate the model’s prediction. The value of AUC can be computed by the trapezoidal rule of integral calculus, as shown in the Equation (10).

(10) $$AUC=\sum_{p=1}^{n}\left(X_{p+1}-X_{p}\right)\times \left(S_{p+1}-S_{p}-S_{p}/2\right)$$

where $X_{p}$ is specificity and $S_{p}$ is sensitivity.

## 3. Results

### 3.1. LSM Acquired by RF Model in the Study Area

Table 4 shows the accuracy of the 10-fold cross-validation of the RF model. The average accuracy of the test dataset of the RF model was 0.907, and Subset 8 had the highest accuracy (0.918) by 10-fold. Hence, the RF model was constructed by using the training dataset of Subset 8.

The trained RF model was applied to the geospatial database to simulate the probability of landslides for each grid in the study area. According to the expert experience method [44], the prediction results of the RF were divided into five classes [68]: very low (<0.06), low (0.06–0.12), medium (0.12–0.21), high (0.21–0.31), and very high (>0.31). Figure 9 and Table 4 show the resulting spatial probability of the landslide distribution maps derived from the RF model. 

**Figure 14:**
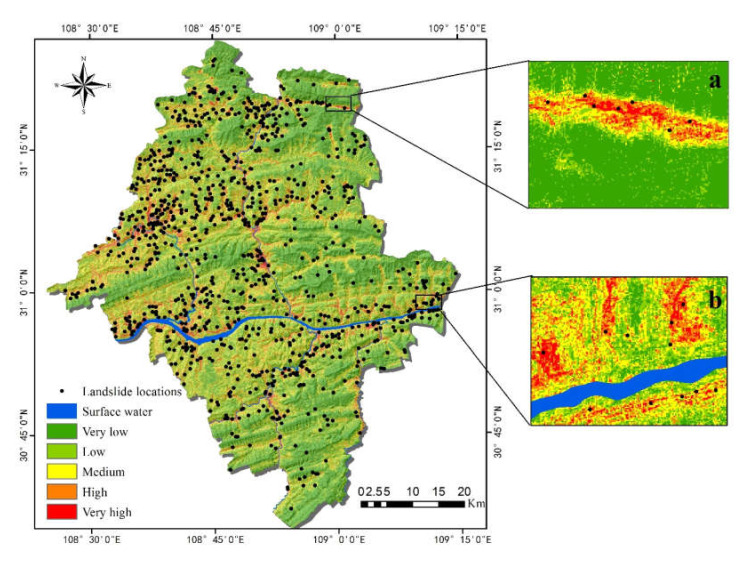
Landslide susceptibility map in the RF model: (**a**) Enlarged area of the valley; (**b**) Enlarged area along the river.

According to the susceptibility evaluation map (Figure 14), most areas of Yunyang County showed low susceptibility and concentrated in relatively flat areas, such as the upper-middle and northeast regions. The areas with high susceptibility to landslides were concentrated on both sides of the Yangtze River and its tributaries, mainly in the southwest and northwest of Yunyang County. Due to scours and soaks of rivers, the soils become loose, extremely prone to landslides under the influence of gravity. On the other hand, within the elevation range of the southwest and northwest parts, the population density is high and human engineering activities are intensive, thus changing the surrounding geological environments and affecting the occurrence of landslide disasters. Moreover, the distribution of areas with high susceptibility to landslides was almost consistent with that of the historical landslide locations (Figure 14a,b).

LSM is a qualitative evaluation of model performance, while statistical analyses (accuracy statistics approach) are with more specific and qualitative features. Table 5 shows the distribution of historical landslides in the five classes. The regions with high and very high susceptibility to landslides accounted for 12.8% of the total area, but 77.5% of the landslides were in these regions. The regions with low and very low susceptibility to landslides accounted for 62.6% of the total area, while only 8.5% of the landslides were in these regions. This means that the landslide locations have a high spatial correlation with the landslide susceptibility. The evaluation shows that the landslide density was increased by approximately 276 times (from 0.021 to 5.806) from very low to very high.

### 3.2. LSM Acquired by the FR Model in Study Area

Based on the analysis of the relationship between the 22 conditioning factors and the landslide occurrence, the application of FR method produced the $\;Fr_{i}\;$ indexes of each class (Table 6). The factors with the most significant correlations with landslide included elevation, slope, lithology, NDVI, annual average rainfall, land cover, distance from roads, and POI kernel density. In particularly, dem showed a negative correlation in all classes, with values of $Fr_{i}$ increasing with dem. The first classes (<690 m) have a $Fr_{i}$ of more than 1, showing a strong correlation to landslides. Concerning slope, the relationship between $Fr_{i}$ and slope is a rule of increasing first and then decreasing. The values from the first three to five classes are more than 1, which indicates that the gentle and incline slope have a greater impact on landslides. The values of $Fr_{i}$ are also strongly correlated with different types of lithology. As the most widely distributed strata in the study area, J2 and J3 have a strong correlation with the $Fr_{i}$ value. NDVI shows a positive correlation in the first four and five classes, which are covered with rich vegetation and play a key role in limiting the occurrence of landslides. The annual average rainfall is the main trigger factor of landslides in the study area. While the number of grids and landslides decreases with increasing rainfall, they have a typical complex correlation with $Fr_{i}$. Concerning land cover, the values of $Fr_{i}$ indicate a positive correlation with ‘Farmland’ and ‘Transportation,’ while ‘Residential land’ has indexes slightly less than 1, possibly indicating an obvious impact of human activities on the occurrence of landslides. Lastly, distance from roads and POI kernel density are two factors of typical human engineering activities. The distance from 0 to 300 m and POI kernel density from the first three to seven classes have a close correlation with landslides. The development and construction in the county in recent years has reduced vegetation covers and damaged slope structures, resulting in an increase of landslides and other natural disasters.

**Figure 15:**
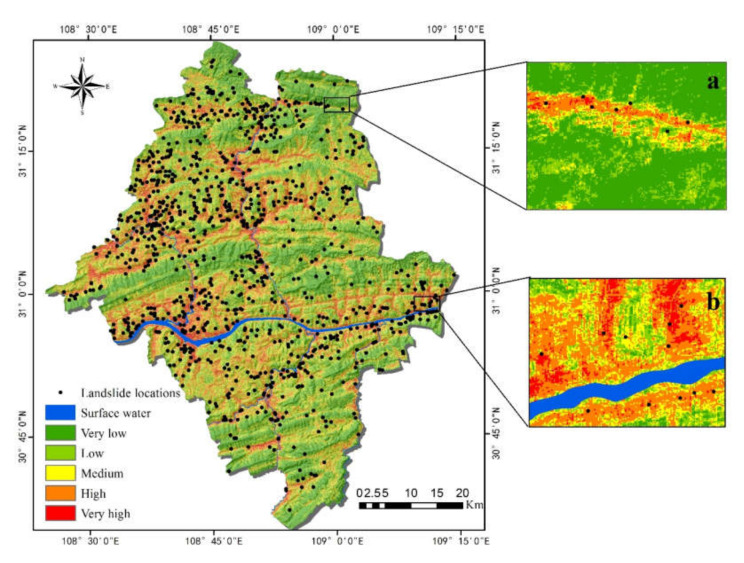
Landslide susceptibility map in the FR model: (**a**) Enlarged area of the valley; (**b**) Enlarged area along the river.

With the help of the FR model, the spatial relations, i.e., LSM, between historical landslides’ locations and contributing factors for the occurrence of landslides were derived (Figure 15). The landslide susceptibility index (LSI) was between 16.50 and 27.99. As in the RF model, based on expert classification, the LSI was divided into five categories: very low (<21.10), low (21.10–22.19), medium (22.19–22.93), high (22.93–24.54), and very high (>24.54).

It can be seen from the susceptibility evaluation map (Figure 15) that most areas of Yunyang County have middle-high susceptibility of landslides, almost wholly distributed on both sides of the Yangtze River, which basically conforms to the historical landslides’ distribution. However, the above distribution pattern is different from the actual landslide distribution in Yunyang County, which mainly expanded the overall landslide susceptibility area compared with the historical landslides’ distribution. 

Moreover, statistical analysis was used to quantitatively evaluate the effectiveness of the model, including the percentage of each susceptibility classification, the number of landslides, the proportion, and density proportion of each category, as shown in Table 7. At the same time, the regions with high and very high susceptibility to landslides in the grading statistics table accounted for 26.7% of the total area, but 52.7% of the landslides were in these regions. The regions with low and very low susceptibility to landslides accounted for 51.6% of the total area, while 24.0% of the landslides were in these regions. The evaluation shows that the landslide density increased by approximately 30 times (from 0.063 to 0.968) as the susceptibility class increased from very low to very high.

### 3.3. Validation and Comparison

Validation is very important for the generated LSI map, so as to evaluate its prediction result reliability. After the landslide susceptibility models were trained and tested, the following four evaluation statistics were used to evaluate the two models: the accuracy, precision, recall rate, and AUC. The results in Table 8 and Table 9 present the confusion matrix of the RF model and the FR model, which were classified by using the library ‘Information Value’ to select a threshold better for the R statistical programming environment instead of the traditional threshold of 0.5. Regarding the accuracy, it can be found that the RF model significantly performed better (0.992), while the accuracy of the FR model was 0.600. Concerning the precision, landslide precision of RF (0.990) was much better than FR (0.147), showing that true landslides account for too few predicted landslides in FR model. On the other hand, non-landslide precision of RF (0.992) was also better than FR (0.954), but both had a similar performance in non-landslides. Because the number of non-landslides in this study is 10 times the landslides, leading to a result that whether for RF or FR, non-landslides were better classified than landslides. The landslide and non-landslide recall rates of RF were both above 0.9, which verify the classification outcomes are rational. For the FR model, the reason why the non-landslide recall rate was relatively low (0.538) is that the areas of high and very high susceptibility were enlarged, resulting in many non-landslides falling in these areas. In fact, compared to the recall rate, the accuracy can better evaluate the predictive capability of a model. Therefore, this study mainly used the accuracy as an evaluation index, while the recall rate was used as a reference.

**Figure 16:**
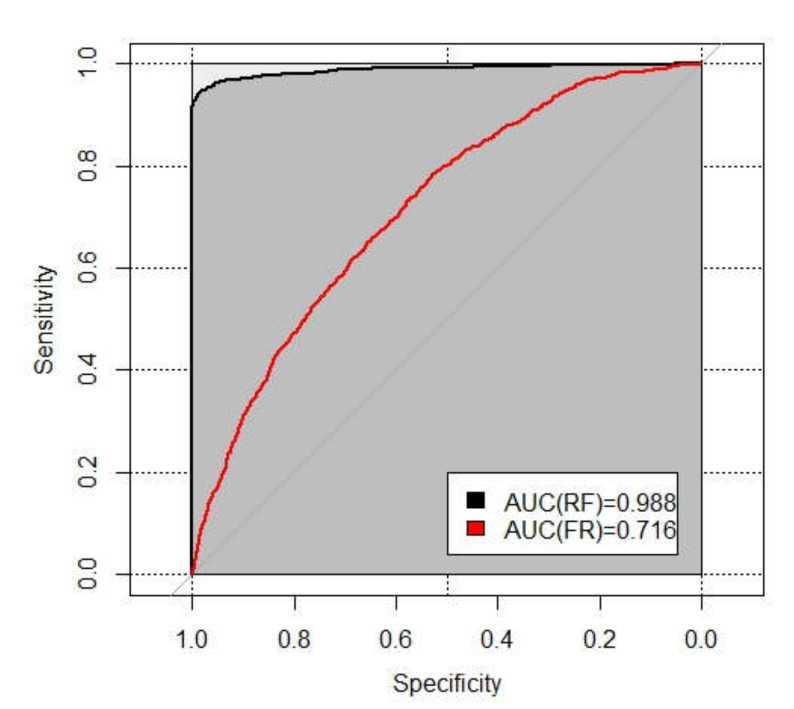
ROC curve and AUC value.

Additionally, this study verified the results of LSM of the two models based on the sample database by performing AUC of ROC. According to Chen et al. [69], an AUC value can be quantified as follows: poor (0.5–0.6), average (0.6–0.7), good (0.7–0.8), very good (0.8–0.9), and excellent (0.9–1). Figure 16 shows the ROC curves of the RF model and the FR model, with AUC values of 0.988 and 0.716, respectively. Both models had an AUC value of more than 0.7 (good and above), so they can be used in analyzing landslide susceptibility of the study area. However, similar to other statistical results, the AUC value for RF was also observed to be better than for FR. Hence, it can be concluded that the predictive performance of RF was better than the FR.

## 4. Discussion

### 4.1. The Comparison of the Two Models

**Figure 17:**
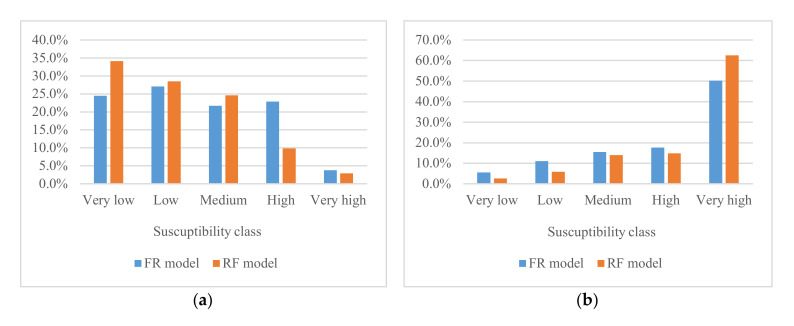
Quantitative comparison of landslide susceptibility class: (**a**) percentage of susceptibility regions (%); (**b**) percentage of landslides (%).

In this study, the RF model and the FR model were selected for a comparative study of landslide susceptibility. The RF model is innovative and has been applied to landslide susceptibility only in recent years, and has shown good performance. The FR model is a traditional method to evaluate landslide susceptibility and can also deliver acceptable results. In this study, a qualitative assessment as shown in Figure 14 and Figure 15 indicates that the results of LSM were similar for both models. Nevertheless, the high and very high regions of FR had obviously larger area than RF, which means that the landslide area is beyond the normal range. This suggests adequate robustness in RF compared with FR. Figure 17 shows the quantitative distribution of landslide susceptibility classes in RF and FR models. Concerning the percentage of susceptibility regions (Figure 17a), the two models have both similarities and differences: the ‘Low’ ‘Medium’ and ‘Very high’ classes of RF and FR are similar, while it is much different in the ‘Very low’ and ‘High’ classes. Consequently, the reason why the performance of the FR was worse than that of the RF is that most areas of Yunyang County were located in very low susceptibility class regions by using the RF model, but low susceptibility class regions by using the FR model. On the other hand, the percentage of susceptibility regions in RF is decreasing as the susceptibility classes goes up (from ‘Very low’ to ‘Very high’), but the FR does not follow this rule. Regarding the percentages of landslides in RF and FR (Figure 17b), they had a positive correlation to the susceptibility class. Except for the class of ‘Very high’ the percentage of landslides of the other four classes of FR are higher than RF’s, resulting in an increased percentage of landslides falling in the four classes of FR. The division of susceptibility regions should follow the guidelines that the density of historical landslides in the low susceptibility area is the smallest, while it is the largest in the high susceptibility area; thus, LSM is consistent with the actual situation of the distribution of historical landslides, which is the basis of expert experience. Finally, the percentage of landslides and percentage of susceptibility regions show a clear negative correlation.

The accuracy, precision, recall rate, and AUC were used to evaluate and compare the capability of the two models. The results showed that the four indexes of the RF model were higher than the FR model. These results may suggest that the conventional method limited the capability for landslide prediction. Such results are similar to the related study [70] that the FR is not highly selective in classification in the study area. This limitation is due to the fact the FR is realized by summing for each cell $Fr_{i}$ obtained considering a causative factor at the same time, while the RF can balance errors for unbalanced data sets, and it can deal with classification and regression problems well. Nevertheless, Hong et al. [71] focused on using four methods to evaluate and compare landslide susceptibility, finding that the AUC value of FR (0.8134) is higher than the AUC value of RF (0.7172). This difference can be explained by the fact that they have small sample sizes (a total of 163 landslides events), but RF operates by constructing a multitude of decision trees; thus, it needs enough samples to perform better.

The results showed that the prediction accuracy of landslides in RF was significantly higher than in the FR model, suggesting that machine learning is usually a good complement to the statistical method when the research issues need special attention to predictability. Secondly, when the sample size is large, the prediction ability of the machine learning method will be greatly promoted. In addition, another reason for better performance of RF than FR model is that the RF has been optimized for two important parameters (mtry and ntree) and the 10-fold cross-validation, while the FR model has not been optimized. For this problem, Guo et al. [72] combined the FR model and the logistic regression model to improve the accuracy of landslide susceptibility evaluation by 4–9%. Subsequent research will need to consider the coupling model to improve the performance for landslide susceptibility.

### 4.2. Distribution Characteristics of New Landslide Events

**Figure 18:**
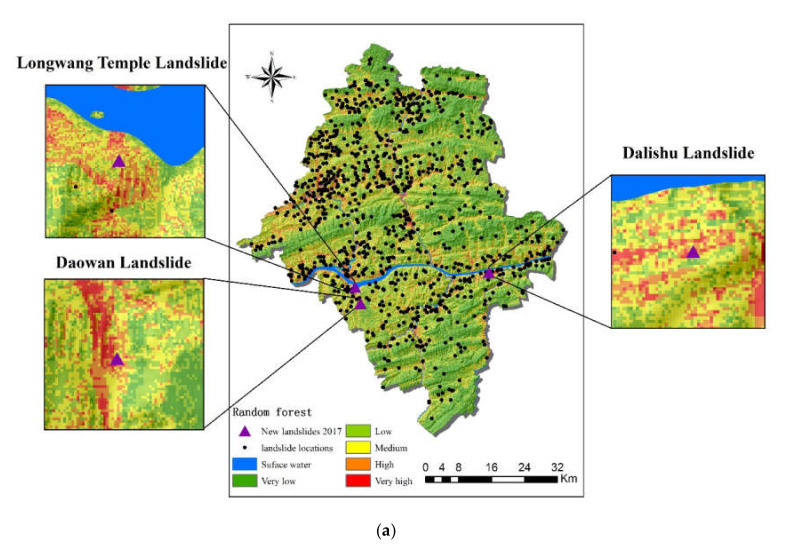

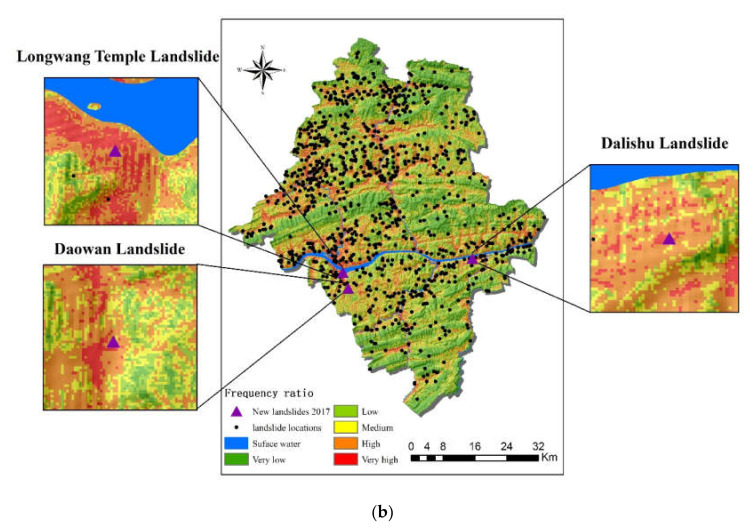
New landslide maps: (**a**) RF; (**b**) FR.

The new landslide distribution map was used as another method to evaluate the accuracy of the models [73]. Information on three new landslides in 2017 was collected in the region. After projecting the locations’ coordinates to landslide susceptibility of the two models, it was shown that all the new landslides were in high or very high susceptibility regions (Figure 18a,b).

The cases of the three new landslides were analyzed to compare the two models: (a) the Longwang Temple Landslide in Panlong Street, Yunyang County. This occurred on 14 September 2017, a typical rainy season in Yunyang County. Like most landslides in the County, the type of the new landslide was soil-related and was induced by precipitation. Concerning the susceptibility mapping of the RF, this new landslide fell in medium and high susceptibility regions, while it only fell in very high susceptibility region in FR. (b) The Daowan Landslide was a soil landslide located in Lao Cao Town, Yunyang County, and occurred on 12 August 2017. Figure 18 shows that the Daowan Landslide was in the saddles of two mountains and occurred during the erosion of rainfall. Whether in RF or FR, this new landslide fell in high or very high susceptibility areas. (c) The Dalishu soil landslide occurred on 6 September 2017. Like the two new landslides above, it was caused by rainfall and fell in high or very high susceptibility region in RF and FR models.

The three new landslides’ locations were compared and analyzed in the two models. Longwang Temple Landslide was in medium and high susceptibility areas, while Daowan Landslide and Dalishu Landslide were in high or very high susceptibility areas. The above results indicated that both the RF model and the FR model had an acceptable prediction accuracy.

### 4.3. Importance of Contributing Factors

**Figure 19:**
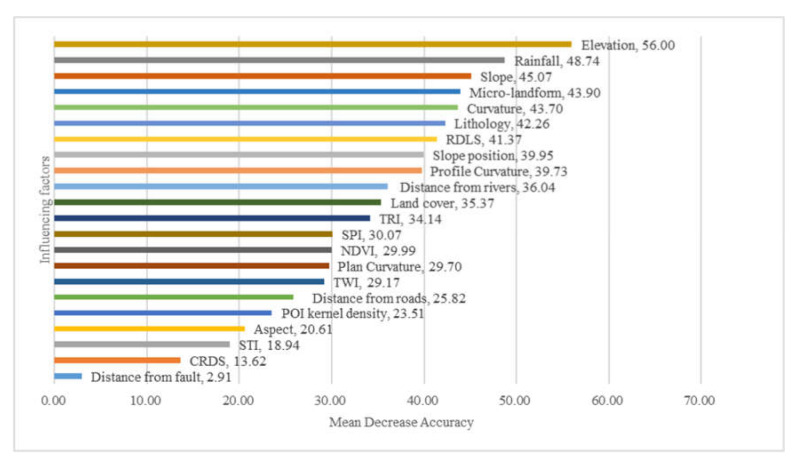
Mean decrease accuracy (sorted in descending order from top to bottom) of attributes, as assigned by the RF.

**Figure 20:**
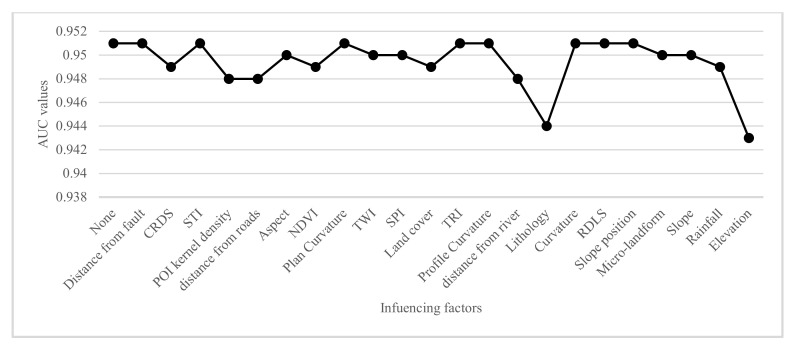
AUC values of RF model with different reduced landslide influencing factors.

Effective and contributing factors play an important role in affecting the prediction accuracy of landslide susceptibility [74]. For this reason, the RF model can give the importance of ranking the landslide factors by the mean decrease accuracy [44]. The importance of each evaluation factor and its impact on the landslide susceptibility are different. Therefore, analyses of the importance and impact of the factors can provide important guidance for landslide disaster prediction and prevention. R studio software (https://rstudio.com/) was used to calculate the mean decrease accuracy of each factor, i.e., changing the order of the factors, and then analyzing the reduction degree of the accuracy of prediction in the RF model after disorder. The larger the value, the greater the significance of the factor. However, when the variables contain noise and correlation, it will affect the importance of ranking results. If the importance ranking is made only once, the result is often inaccurate [75]. Therefore, this study used the averages of 10 times as the final ranking result (Figure 19). On the other hand, we retained the model by reducing each of the 22 factors in turn. Figure 20 shows the AUC value for each retraining. The above two methods were combined to evaluate the importance of factors.

**Figure 21:**
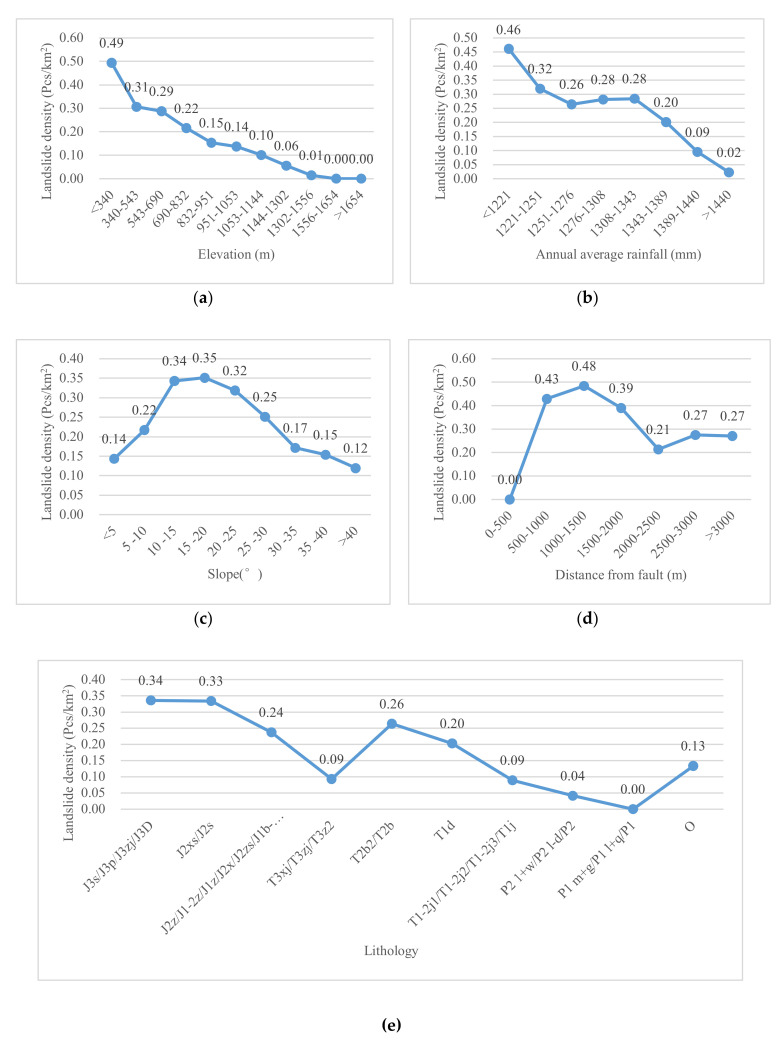
Typical factors in landslide density statistics: (**a**) Elevation; (**b**) Annual average rainfall; (**c**) Slope; (**d**) Distance from faults; (**e**) Lithology.

Figure 19 shows that the elevation, annual average rainfall, and slope are the top three factors affecting the susceptibility of landslides in Yunyang County, with a mean decrease accuracy of 56.00, 48.74, and 45.07, respectively, while the most insignificant factor was the distance from faults, with a mean decrease accuracy of 2.91. Similarly, Figure 20 shows that the elevation had the greatest influence on the AUC value of RF model, followed by lithology. Secondly, if POI kernel density, distance from roads and distance from rivers are removed, the predictive ability of the RF model will be reduced by 0.003. Although the POI kernel density, an innovation factor in this study, was lower in the ranking of the factors, it contributes to the prediction accuracy of the model. Furthermore, they were also similar to the typical factors in the FR model, and the key to affect the occurrence of landslides in Yunyang County. In order to better analyze the relationship between factors and landslide, a statistical map of historical landslide density ranking of the four types was drawn: elevation, annual average rainfall, slope, and lithology (Figure 21a–d). In contrast, the distance from fault was the least significant factor (Figure 21e). The abscissa corresponds to the factor value class, and the ordinate corresponds to the landslide density. Thus, the larger the ordinate value is, the easier a landslide occurs.

It can be seen from Figure 21a that the landslide density had a negative correlation with the elevation: the landslide density is higher at lower elevations. Yunyang County sits in a typical mountainous environment, with a large elevation difference and a low elevation. The areas at lower elevations have sparse vegetation coverage and loose soils. Most of such areas are in the watershed regions, with heavy human engineering activities; therefore, landslides occur frequently. The areas at higher elevation have thick vegetation coverage and few human activities; therefore, fewer landslides occur. Secondly, Figure 21b shows that the landslide density had a negative correlation with the average rainfall over the years. Rainfall is the main cause of landslides in the study area. Especially in the monsoon seasons, a scouring effect is imposed on the slope surface, causing unstable rocks and soil particles on slope surfaces to be carried away by surface runoff formed by rainfall. Erosion usually occurs in the slope body, and the average rainfall over many years also affects the development of vegetation, which in turn affects the development of landslides. However, the annual average rainfall is the average of the rainfall for many years, and it is different from short-term rainfall, which includes antecedent rainfall and rainfall on the day of a landslide. On the one hand, the annual average rainfall not only affects the slope itself, but also affects the development of vegetation. Generally, the more the rainfall, the lusher the vegetation, thereby reducing the possibility of landslides. On the other hand, generally, the areas with the most precipitation are in the middle of mountains, and the precipitation will decrease significantly near the top. Human activities are more significant in the low and middle mountains, which may affect the occurrence of landslides greatly. The landslide density and slope showed a typical normal distribution relationship (Figure 21c). The literature review revealed that a single conditioning factor such as slope may not necessarily always have high importance in landslide susceptibility evaluation [76]. However, the slope still plays a role in the landslide susceptibility model for Yunyang County. Landslides may be induced by a steep slope and varying shear stresses that is external forces affect deformation and sliding relatively of landslide body [77]. On the other hand, gentle slopes are expected to have a low frequency of landslides, because of the common lower shear stresses that are associated with low gradients. Lithology is a kind of categorical variable; J2 and J3 are the most widely distributed strata in the study area, and a high landslide density appear with them (Figure 21e). The distance to faults is of the lowest importance (Figure 21d), and there was no obvious correlation between the landslide density and faults. This is related to the fact that there is only one small fault in Yunyang County. Wen et al. [78] showed that faults have little effect on landslides, except for the strong earthquakes area.

## 5. Conclusions

LSM provides the possibility of occurrence of landslides and is a useful tool for the prevention and evaluation of landslides. This study took Yunyang County, a typical and severely-affected area in the Chongqing Section of Three Gorges Reservoir, as a research case, and employed the RF model and the FR model to conduct a comparative study of LSM. The main conclusions are as follows:

(1) A total of 987 historical landslides are identified with landslide susceptibility inventory, which contains the historical records, satellite images, and extensive field surveys, and 94.7% of the landslides are soil landslides, while 84.8% are induced by rainfall. Subsequently, 70% of the landslides were used as the training dataset and 30% as the testing dataset. Twenty-two factors in five categories, including elevation, slope, slope position, aspect, and lithology, were selected as the contributing factors of landslides in Yunyang County. By optimizing two important parameters of RF, with 10-fold-cross validation for the best sample on R software, a more efficient RF model can be built to evaluate landslide susceptibility. As a result, the LSM was produced with the two models.
(2) In mapping evaluation, the RF model had 77.5% of historical landslides falling in the regions with high or very high susceptibility, accounting for about 12.8% of the total area. The regions with low or very low susceptibility to landslides accounted for 62.6% of the total area, while only 8.5% of landslides were in these areas. On the other hand, the FR model had 52.7% of the landslide falling in the high or very high susceptibility regions, accounting for of 26.7% of the total area. The regions with very low or low susceptibility accounted for 51.6% of the total area, while 24.0% of the landslides were in these areas. The AUC values under the ROC curve of the RF model and the FR model were 0.988 and 0.716, respectively. Similarly, accuracy, precision, and recall ratio of RF were higher than FR. Furthermore, in high and very low classes, RF performed better. In addition, the susceptibility mapping results of the two models both had a high spatial correlation with new landslides in 2017. The evaluation results above show that the RF model has higher accuracy, reliability, and stability. The RF model is more suitable for landslide susceptibility evaluation in Yunyang County than the FR model. The performance of models depends not only on algorithms, but also on the specific conditions of the study areas and the selection of impacting factors. Therefore, this study cannot conclude that the RF model is definitely the best. Compared with the FR model, the RF model has higher prediction accuracy. This finding is similar to the results of Sun et al. [73], who used RF to study Fengjie County (a neighbor of Yunyang County, with a similar geographic environment).
(3) Finally, the importance-ranking results obtained from the impact factor importance analysis and AUC values of RF model with different reduced landslide influencing factors are in accordance with the basic laws of the geology and consistent with previous research findings. They can provide guidance for landslide management. The elevation, annual average rainfall, slope, lithology, POI kernel density, distance from roads, and distance from rivers were the main important landslide contributors in Yunyang County, while the contribution rate of faults was the smallest. In particular, as the highlight of this study, the POI kernel density proves useful in landslide susceptibility models. There are complex relationships between the factors, and the occurrence of landslides is inseparable from the combined effects of human and natural factors.

## Figures and Tables

**Table 1:** Classification of conditioning factors.

Factor	Type	Classification
Elevation/m	Continuous	(1) <340; (2) 340~543; (3) 543~690; (4) 690~832; (5) 832~951; (6) 951~1053; (7) 1053~1144; (8) 1144~1302; (9) 1302~1556; (10) 1556~1654; (11) >1654
Slope/°	Continuous	(1) <5; (2) 5~10; (3) 10~15; (4) 15~20; (5) 20~25; (6) 25~30; (7) 30~35; (8) 35~40; (9) >40
RDLS/m	Continuous	(1) <20; (2) 20~30; (3) 30~40; (4) 40~50; (5) 50~80; (6) 80~120; (7) >120
Aspect	Categorical	(1) Flat; (2) North; (3) Northeast; (4) East; (5) Southeast; (6) South; (7) Southwest; (8) West; (9) Northwest
Slope position	Categorical	(1) Ridge; (2) Upper slope; (3) Middle slope; (4) Flats slope; (5) Lower slope; (6) Valley
Micro-landform	Categorical	(1) Canyons, and Deeply incised streams; (2) Midslope drainages, and shallow valleys; (3) Upland drainages, and Headwaters; (4) U-shape valleys; (5) Plains; (6) Open slopes; (7) Upper slopes, and Plateau; (8) Local ridges hills in valleys; (9) Midslope ridges, and Small hills in plains; (10) Mountain tops, and High narrow ridges
Curvature	Continuous	(1) <−1; (2) −1~−0.5; (3) −0.5~0; (4) 0~0.5; (5) 0.5~1; (6) >1
Profile Curvature	Continuous	(1) <−1; (2) −1~−0.5; (3) −0.5~0; (4) 0~0.5; (5) 0.5~1; (6) >1
Plan Curvature	Continuous	(1) <−1; (2) −1~−0.5; (3) −0.5~0; (4) 0~0.5; (5) 0.5~1; (6) >1
TRI	Continuous	(1) <1.05; (2) 1.05~1.1; (3) 1.1~1.15; (4) 1.15~1.2; (5) >1.2
TWI	Continuous	(1) <4; (2) 4~6; (3) 6~8; (4) 8~10; (5) >10
STI	Continuous	(1) <20; (2) 20~40; (3) 40~70; (4) 70~100; (5) 100~200; (6) >200
SPI	Continuous	(1) <15; (2) 15~30; (3) 30~45; (4) 45~60; (5) 60~100; (6) 100~1000; (7) >1000
Lithology	Categorical	(1) J3s, J3p, J3zj, J3D; (2) J2xs, J2s; (3) J2z, J1-2z, J1z, J2x, J2zs, J1b-j2Q; (4) T3xj, T3zj, T3z2; (5) T2b2, T2b; (6) T1d; (7) T1-2j1, T1-2j2, T1-2j3, T1j; (8) P2 1+w, P2 l-d, P2; (9) P1 m + g, P1 l + q, P1, C; (10) O
Distance from fault/m	Continuous	(1) <500; (2) 50~1000; (3) 1000~1500; (4) 1500~2000; (5) 2000~2500; (6) 2500~3000; (7) >3000
CRDS	Categorical	(1) Dip-slope I; (2) Dip-slope II; (3) Outward slope; (4) Oblique slope; (5) Tangential slope; (6) Reverse slope; (7) Flat
NDVI	Continuous	(1) 0~0.1; (2) 0.1~0.15; (3) 0.15~0.2; (4) 0.2~0.25; (5) >0.25
Distance from rivers/m	Continuous	(1) <100; (2) 100~200; (3) 200~300; (4) 300~400; (5) 400~500; (6) 500~600; (7) >600
Annual average rainfall/mm	Continuous	(1) <1221; (2) 122~1251; (3) 1251~1276; (4) 1276~1308; (5) 1308~1343; (6) 1343~1389; (7) 1389~1440; (8) >1440
Land cover	Categorical	(1) Meadow; (2) Farmland; (3) Water area; (4) Forest; (5) Garden plot; (6) Others/14531/0.0000; (7) Residential land; (8) Transportation
Distance from roads/m	Continuous	(1) <100; (2) 100~200; (3) 200~300; (4) 300~400; (5) 400~500; (6) 500~600; (7) >600
POI kernel density	Continuous	(1) 0–1; (2) 1–2; (3) 2–3; (4) 3–4; (5) 4–5; (6) 5–10; (7) >10

**Table 2:** Data and data sources.

Data Name	Data Sources	Type	Scale
Historical landslide	Chongqing Geological Monitoring Station	Dataset	
Elevation	Aster satellite	Grid	30 m
Geological data	National Geological Data Center	Grid	1:200,000
Land cover	Chongqing Municipal Bureau of Land and Resources	Vector	1:100,000
Administrative division	Chongqing Municipal Bureau of Land and Resources	Vector	1:100,000
River network	Chongqing Water Resources Bureau	Vector	1:100,000
Satellite image	Geospatial Data Cloud platform	Grid	30 m
Annual rainfall	Chongqing Meteorological Administration	Dataset	90 m
Road	Chongqing Transportation Commission	Vector	1:100,000
POI of Chongqing	Web Crawler	Dataset	

**Table 3:** Explanation of statistical-index-based evaluations.

No	Metric	Equation	Definition
1	Precision	$Precision=\frac{TP}{TP+FP}$	The fraction of relevant instances in the retrieved instances.
2	Sensitivity (SST)	$SST=\frac{TP}{TP+FN}$	The percentage of landslide cells that are correctly classified.
3	Specificity (SPF)	$SPF=\frac{TN}{TN+FP}$	The percentage of non-landslide cells that are correctly classified.
4	Accuracy (ACC)	$ACC=\frac{TP+TN}{M}$	The proportion of landslide and non-landslide cells which are correctly classified.
5	Recall	$Recall=\frac{TP}{TP+FN}$	It indicates how many positive examples in the sample are predicted correctly.

TP is the number of correctly predicted landslide cells. FP is the sum of cells of non-landslides that are classified as landslide. FN is the sum of cells of landslides that are classified as non-landslide. TN is the number of correctly predicted non-landslide cells. M is the sum of landslides and non-landslides.

**Table 4:** The accuracy of 10-fold cross-validation of the RF model.

Subset	Accuracy		Subset	Accuracy	
	Training	Testing		Training	Testing
1	1.000	0.900	6	1.000	0.902
2	1.000	0.904	7	1.000	0.906
3	1.000	0.909	8	1.000	0.918
4	1.000	0.915	9	1.000	0.885
5	1.000	0.916	10	1.000	0.916

**Table 5:** Statistic result of landslide susceptibility in different classes of RF.

Landslide Probability	Susceptibility Class	Grid Number	Area Proportion	Landslide	Landslide Proportion	Density Proportion (Pcs/km^2^)
<0.06	Very low	1,373,501	34.1%	26	2.6%	0.021
0.06–0.12	Low	1,147,495	28.5%	58	5.9%	0.056
0.12–0.21	Medium	991,347	24.6%	138	14.0%	0.155
0.21–0.31	High	396,368	9.8%	147	14.9%	0.412
>0.31	Very high	118,277	2.9%	618	62.6%	5.806

**Table 6:** Classification and FR of conditioning factors.

Factor	Type	Classification/Grid Number/FR
Elevation/m	Continuous	(1) <340/647744/1.8208; (2) 340~543/99121/1.1279; (3) 543~690/783868/1.0606; (4) 690~832/614750/0.7927; (5) 832~951/390895/0.5657; (6) 951~1053/235266/0.5048; (7) 1053~1144/142794/0.3728; (8) 1144~1302/140356/0.2042; (9) 1302~1556/75487/0.0543; (10) 1556~1654/13725/0.0000; (11) >1654/5892/0.0000
Slope/°	Continuous	(1) <5/231687/0.5303; (2) 5~10/562181/0.8013 ; (3) 10~15/747696/1.2652; (4) 15~20/743059/1.2952; (5) 20~25/638172/1.1743; (6) 25~30/481841/0.9264; (7) 30~35/317712/0.6316; (8) 35~40/180510/0.5672; (9) >40/139136/0.4415
RDLS/m	Continuous	(1) <20/1498140/1.0157; (2) 20~30/976617/1.3054; (3) 30~40/708930/1.0616; (4) 40~50/433899/0.6350; (5) 50~80/397593/0.5689; (6) 80~120/40815/0.2015; (7) >120/3177/0.0000
Aspect	Categorical	(1) Flat/832/0.0000; (2) North/559138/1.0693; (3) Northeast/418623/0.8413; (4) East/476389/0.9542; (5) Southeast/493071/1.0382; (6) South/599652/0.9629; (7) Southwest/493884/1.1028 ; (8) West/501655/1.0776; (9) Northwest/498750/0.9278
Slope position	Categorical	(1) Ridge/1487219/1.0188; (2) Upper slope/282489/1.0583; (3) Middle slope/74587/0.4941; (4) Flats slope/469978/1.0631; (5) Lower slope/217136/0.9619; (6) Valley/1510585/0.9814
Micro-landform	Categorical	(1) Canyons, and Deeply incised streams/1447542/1.0920; (2) Midslope drainages, and shallow valleys/93339/1.0530; (3) Upland drainages, and Headwaters/213973/0.6507; (4) U-shape valleys/269975/1.3197; (5) Plains/14080/0.0000; (6) Open slopes/67096/1.2207; (7) Upper slopes, and Plateau/242500/0.8444; (8) Local ridges hills in valleys/214246/0.9940; (9) Midslope ridges, and Small hills in plains/99198/1.8165; (10) Mountain tops, and High narrow ridges/1380045/0.8606
Curvature	Continuous	(1) <−1/739914/0.9354; (2) −1~−0.5/473674/0.9597; (3) −0.5~0/932464/1.2473; (4) 0~0.5/697900/0.9213; (5) 0.5~1/455266/0.9355; (6) >1/742776/0.8932
Profile Curvature	Continuous	(1) <−1/397552/0.8344; (2) −1~−0.5/466912/1.0087; (3) −0.5~0/1127645/0.9914; (4) 0~0.5/1146502/1.0787; (5) 0.5~1/495095/1.0836; (6) >1/408288/0.8526
Plan Curvature	Continuous	(1) <−1/199130/0.7198; (2) −1~−0.5/399120/0.9440; (3) −0.5~0/1453089/1.0963; (4) 0~0.5/1352014/1.0329; (5) 0.5~1/421528/0.8549; (6) >1/217113/0.7922
TRI	Continuous	(1) <1.05/1956974/1.0568; (2) 1.05~1.1/921054/1.1916; (3) 1.1~1.15/495536/0.9917; (4) 1.15~1.2/273264/0.6744; (5) >1.2/395166/0.5078
TWI	Continuous	(1) <4/522384/0.7134; (2) 4~6/2078046/1.0327; (3) 6~8/878720/1.0346; (4) 8~10/368110/1.1904; (5) >10/194734/0.9043
STI	Continuous	(1) <20/2917053/0.9841; (2) 20~40/522250/0.9410; (3) 40~70/282523/0.9857; (4) 70~100/119897/1.3321; (5) 100~200/119007/1.3076; (6) >200/81264/1.0583
SPI	Continuous	(1) <15/1742958/1.0315; (2) 15~30/660771/0.8987; (3) 30~45/276316/0.9485; (4) 45~60/166651/0.9092; (5) 60~100/256386/0.7986; (6) 100~1000/734492/1.0649; (7) >1000/204420/1.2220
Lithology	Categorical	(1) J3s, J3p, J3zj, J3D/1151138/1.2333; (2) J2xs, J2s/1168089/1.2294; (3) J2z, J1-2z, J1z, J2x, J2zs, J1b-j2Q/617821/0.8741; (4) T3xj, T3zj, T3z2/359056/0.3418; (5) T2b2, T2b/379171/0.9711; (6) T1d/87456/0.7485; (7) T1-2j1, T1-2j2, T1-2j3, T1j/211999/0.3281; (8) P2 1+w, P2 l-d, P2/53807/0.1521; (9) P1 m + g, P1 l + q, P1, C/1323/0.0000; (10) O/8310/0.4923
Distance from fault/m	Continuous	(1) <500/3370/0.0000; (2) 50~1000/517/1.5808; (3) 1000~1500/6879/1.7852; (4) 1500~2000/8551/1.4362; (5) 2000~2500/10401/0.7871;(6) 2500~3000/12126/1.0127; (7) >3000/3993800/0.9983
CRDS	Categorical	(1) Dip-slope I/65686/1.5565; (2) Dip-slope II/280703/1.0053; (3) Outward slope/345779/1.2419; (4) Oblique slope/1001302/1.0987; (5) Tangential slope/635257/1.1330; (6) Reverse slope/1476082/0.8672; (7) Flat/231650/0.5296
NDVI	Continuous	(1) 0~0.1/208363/0.6878; (2) 0.1~0.15/390391/0.7762; (3) 0.15~0.2/1282355/0.9196; (4) 0.2~0.25/1429333/1.0370; (5) >0.25/731025/1.2771
Distance from rivers/m	Continuous	(1) <100/244170/0.8718; (2) 100~200/203437/1.7104; (3) 200~300/214861/1.6194; (4) 300~400/186399/1.6471; (5) 400~500/192759/1.2317; (6) 500~600/182581/1.1210; (7) >600/2816099/0.8460
Annual average rainfall/mm	Continuous	(1) <1221/291710/1.6968; (2) 122~1251/521092/1.1775; (3) 1251~1276/626074/0.9735; (4) 1276~1308/854004/1.0346; (5) 1308~1343/832774/1.0463; (6) 1343~1389/663280/0.7401; (7) 1389~1440/199205/0.3491; (8) >1440/49295/0.0830
Land cover	Categorical	(1) Meadow/727009/0.8346; (2) Farmland/71553/1.4188; (3) Water area/3057711/0.7712; (4) Forest/9809/0.9130; (5) Garden plot/26616/0.8581; (6) Others/14531/0.0000; (7) Residential land/132682/0.9227; (8) Transportation/109/1.4084
Distance from roads/m	Continuous	(1) <100/625582/1.7864; (2) 100~200/424193/1.0905; (3) 200~300/402164/1.2113; (4) 300~400/313648/0.9266; (5) 400~500/298588/0.7129; (6) 500~600/258887/0.9171; (7) >600/1717244/0.7175
POI kernel density	Continuous	(1) 0–1/286078/0.5581; (2) 1–2/1291095/0.8624; (3) 2–3/1153628/1.0752; (4) 3–4/513828/1.2508; (5) 4–5/244087/0.8553; (6) 5–10/316584/1.1508; (7) >10/235006/1.3238;

**Table 7:** Statistic result of landslide susceptibility in different classes of FR.

Landslide Probability	Susceptibility Class	Grid Number	Area Proportion	Landslide	Landslide Proportion	Density Proportion (Pcs/km^2^)
<21.10	Very low	986,559	24.5%	56	5.7%	0.063
21.10–22.19	Low	1,093,869	27.1%	181	18.3%	0.184
22.19–22.93	Medium	875,313	21.7%	230	23.3%	0.292
22.93–24.54	High	924,737	22.9%	387	39.2%	0.465
>24.54	Very high	152,601	3.8%	133	13.5%	0.968

**Table 8:** Confusion matrix of RF.

RF		True Condition		Summation
		Landslide	Non-Landslide	
**Prediction** **Condition**	**Landslide**	907	9	Precision: 0.990
	**Non-landslide**	80	9861	Precision: 0.992
**Summation**		Recall: 0.919	Recall: 0.999	Accuracy: 0.992

**Table 9:** Confusion matrix of FR.

FR		True Condition		Summation
		Landslide	Non-Landslide	
**Prediction** **Condition**	**Landslide**	711	4114	Precision: 0.147
	**Non-landslide**	276	5756	Precision: 0.954
**Summation**		Recall: 0.720	Recall: 0.538	Accuracy: 0.600
